# Two-step continuous-flow synthesis of 6-membered cyclic iodonium salts via anodic oxidation

**DOI:** 10.3762/bjoc.19.2

**Published:** 2023-01-03

**Authors:** Julian Spils, Thomas Wirth, Boris J Nachtsheim

**Affiliations:** 1 Institute for Organic and Analytical Chemistry, University of Bremen, Leobener Straße 7, 28359 Bremen, Germany; 2 School of Chemistry, Cardiff University, Park Place, Main Building, Cardiff CF10 3AT, UK

**Keywords:** electrochemistry, flow chemistry, hypervalent compounds, iodine, oxidation

## Abstract

We describe a multi-step continuous-flow procedure for the generation of six-membered diaryliodonium salts. The accompanying scalability and atom economy are significant improvements to existing batch methods. Benzyl acetates are submitted to this two-step procedure as highly available and cheap starting materials. An acid-catalyzed Friedel–Crafts alkylation followed by an anodic oxidative cyclization yielded a defined set of cyclic iodonium salts in a highly substrate-dependent yield.

## Introduction

**Scheme 1:**
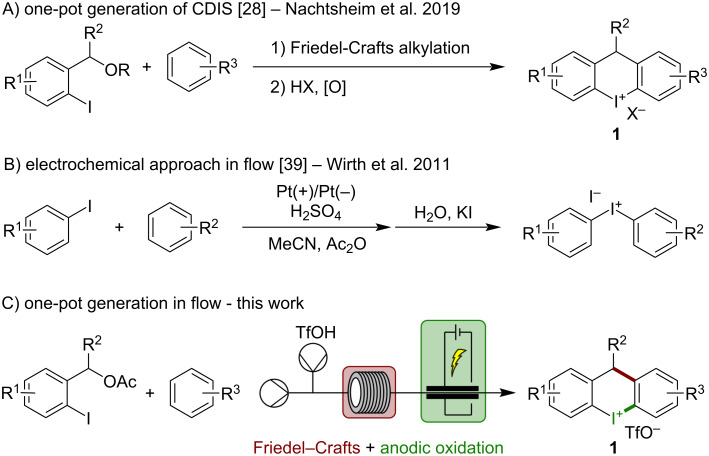
Synthesis of acyclic (DIS) and cyclic (CDIS) diaryliodonium salts.

Hypervalent iodine compounds (HVI) are well-established reagents for synthetic chemists. They are portrayed as an alternative to otherwise hazardous transition metals. This is due to their great reactivity in electrophilic group transfers [1–4], photo- or organocatalysis [5–15], and their utility as building blocks for the synthesis of natural products [16–21]. One subclass of HVIs is diaryliodonium salts (DIS), which have been used as versatile electrophilic arylation reagents in metal-catalyzed and metal-free reactions [22–23]. The corresponding cyclic diaryliodonium salts (CDIS) have also been investigated as useful building blocks for the synthesis of larger diaryl-based molecules, (hetero)aromatic tricyclic systems, or new aryl-moieties [24–25]. Most methods that generate DIS utilize iodoarenes as starting materials [26–27]. In these one-pot procedures, iodoarenes react with nucleophilic arenes under oxidative acidic conditions.The synthesis of CDIS is more challenging since the arene moiety must be covalently connected to the iodoarene prior to cyclization. Recently, we published two new methods that describe the generation of carbon- and heteroatom-bridged CDIS [28–29]. Herein, we improved the formation of iodoarenes through a Brønsted acid-mediated Friedel–Crafts reaction followed by an oxidative cyclization to form the desired CDIS **1** (Scheme 1A). This one-pot approach is based on *ortho*-iodinated benzyl alcohols as starting materials. It allows access to a variety of otherwise tedious to synthesize CDIS robustly in short reaction times. A significant drawback still is the use of stoichiometric amounts of chemical oxidants, which decreases the atom economy and necessitates additional workup procedures. A possible solution is the anodic oxidation of iodoarenes as electrochemistry is a highly economical tool that avoids chemical oxidants for synthesizing hypervalent iodine reagents [30]. Iodoarenes are suitable and well-established mediators in either in- or ex-cell electrochemical processes [31–36].

Nonetheless, HVIs, DIS and CDIS have been generated by anodic oxidation [37–40]. Due to the apparent advantages of electrochemical processes, their implementation in flow is simple and straightforward since further dilution or additives are unnecessary [41–42]. One early example of this combination in the field of HVI chemistry is the anodic oxidation of iodoarenes to form DIS by Wirth et al. (Scheme 1B) [39]. Herein, established conditions for synthesizing DIS were transferred into flow chemistry utilizing a model flow reactor with two platinum electrodes. Other recent examples include the generation of five-membered CDIS utilizing fluorinated alcohols as a solvent [37–38]. Therefore, we attempt to transfer our already established one-pot procedure towards CDIS **1** into a multi-step electrochemical flow process, improving reaction times, atom economy, and scalability (Scheme 1C).

## Results and Discussion

We initially investigated the second step of the proposed multi-step procedure since we assumed it to be the more challenging. We initially intended to oxidize and cyclize the intermediate iodoarene **2** electrochemically under batch conditions (Table 1). Through preliminary observations (see Supporting Information File 1, Table S1), triflic acid was established to be suitable for cyclization and as a counterion. 2,2,2-Trifluoroethanol (TFE) as a solvent already resulted in a yield of 45% of compound **1a** (Table 1, entry 1). It was possible to reduce its amount to 20 vol % by replacement with MeCN or CH_2_Cl_2_ without any significant drop in the yields (40–46%, Table 1, entries 2 and 3). By exchanging TFE for the more stabilizing 1,1,1,3,3,3-hexafluoropropan-2-ol (HFIP) it was possible to increase the yield even further to 78% (Table 1, entry 4) [43–44]. Finally, we decreased the amount of TfOH to 2 equiv still yielding product **1a** in 76% yield (Table 1, entry 5).

**Table 1:** Optimization of the oxidation and cyclization in batch.

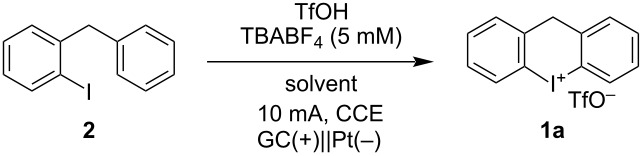				

Entry^a^	TfOH [equiv]	Solvent	Charge [F]	**1a** Yield [%]

1	5	TFE	2.1	45
2	5	**MeCN**/TFE (4:1)	2.0	40
3	5	**CH_2_Cl_2_**/TFE (4:1)	2.2	46
**4**	**5**	**CH** **_2_** **Cl** **_2_** **/HFIP (4:1)**	**2.0**	**78**
5	**2**	CH_2_Cl_2_/HFIP (4:1)	2.0	76

^a^Electrolysis was carried out with an ElectraSyn 2.0 in an undivided cell. Vials were equipped with a glassy carbon (GC) anode and a Pt cathode. Immersed area: 2.4 cm^2^. General reaction conditions: **2** (0.200 mmol), TfOH, TBABF_4_ (0.005 M), solvent (40.0 mM, 5 mL), 10 mA, CCE.

After establishing the optimized reaction conditions for batch, we wanted to find conditions for a flow procedure. It was observed that plastic parts made out of PEEK did not tolerate the acidic conditions in HFIP, and the solvent was changed back to TFE. This worked surprisingly well with only two equivalents of TfOH forming product **1a** in 74% yield (Table 2, entry 1). We found that these reaction conditions cannot be applied in the proposed multi-step procedure since the added benzene clogs the flow reactor through precipitation of a black solid.

**Table 2:** Optimization of the oxidation and cyclization in flow.

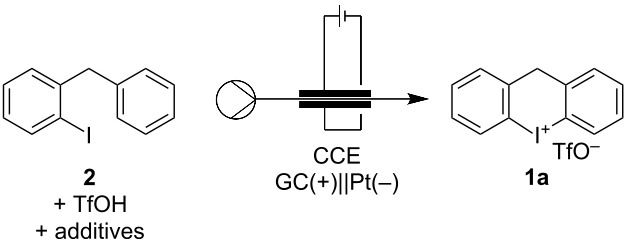					

Entry^a^	TfOH [equiv]	Additives	Solvent	Current [mA]	**1a** Yield [%]

1	2	TBABF_4_	CH_2_Cl_2_/TFE	32 (2.0 F)	74
2	2	TBABF_4_ PhH (9 equiv)	**MeNO** **_2_**	32 (2.0 F)	48
3	2	TBABF_4_ PhH (9 equiv)	MeNO_2_/**TFE**	32 (2.0 F)	63
**4**	**2**	**PhH (9 equiv)**	**MeNO** **_2_** **/TFE**	**32 (2.0 F)**	**62**
5	2	PhH (9 equiv)	MeNO_2_/TFE	**48 (3.0 F)**	62

^a^Reaction was performed in a Vapourtec Ion electrochemical flow reactor with a glassy carbon (GC) anode and a Pt cathode. General reaction conditions: **1** (0.1 M), TBABF_4_ (0.005 M). Flowrate: 0.1 mL·min^−1^. Yield is based on collecting for 20 min (0.200 mmol) after two reactor volumes had passed at the respective conditions.

We could overcome this issue by employing MeNO_2_ as a solvent, which leads to a 48% yield of **1a** (Table 2, entry 2). This yield could be increased further by a combination with TFE to 63% (Table 2, entry 3). We could also omit the additional electrolyte TBABF_4_ with no product loss (Table 2, entry 4), while the yield could not be improved further by increasing the charge (Table 2, entry 5).

For a final combination of the two reaction steps, benzyl acetate **3a** was chosen as a model substrate due to its high solubility and good reactivity as was previously demonstrated by us (Table 3) [28]. We determined an optimal reaction time of 20 min for the Friedel–Crafts reaction via GC analysis. Both steps in series resulted in successful product formation with an albeit slightly diminished yield of 49% (Table 3, entry 3).

**Table 3:** Optimization of the two-step flow process in series.

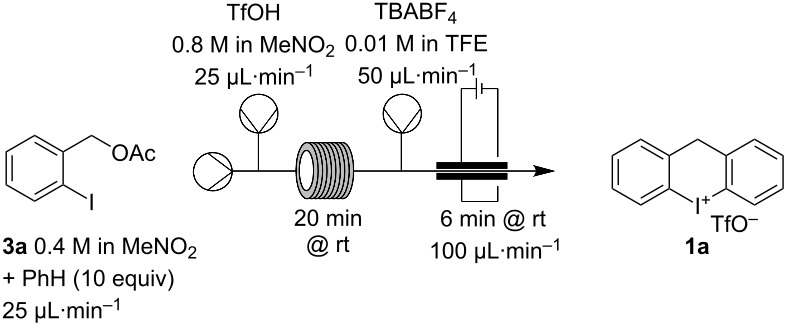			

Entry^a^	Electrolyte	Current [mA]	**1a** Yield [%]

1	–	32 (2.0 F)	49
**2**	**TBABF** **_4_**	**32 (2.0 F)**	**54**
3	TBABF_4_	**48 (3.0 F)**	54

^a^Reaction was performed in a Vapourtec Ion electrochemical flow reactor with a glassy carbon (GC) anode and a Pt cathode. Yield is based on collecting for 20 min (0.200 mmol) after two reactor volumes had passed at the respective conditions.

We increased the yield to 54% by introducing an electrolyte (Table 3, entry 2). A further current increase had no beneficial effect (Table 3, entry 3). We then investigated longer reaction times (4 × 50 min, 4 × 0.500 mmol), but without additional electrolyte the overall yield of 38% was significantly lower. It was possible to compensate for this to some degree by using TBABF_4_ as shown in Table 3, entry 2. This adjustment improved the overall yield to 43%.

**Figure 1:**
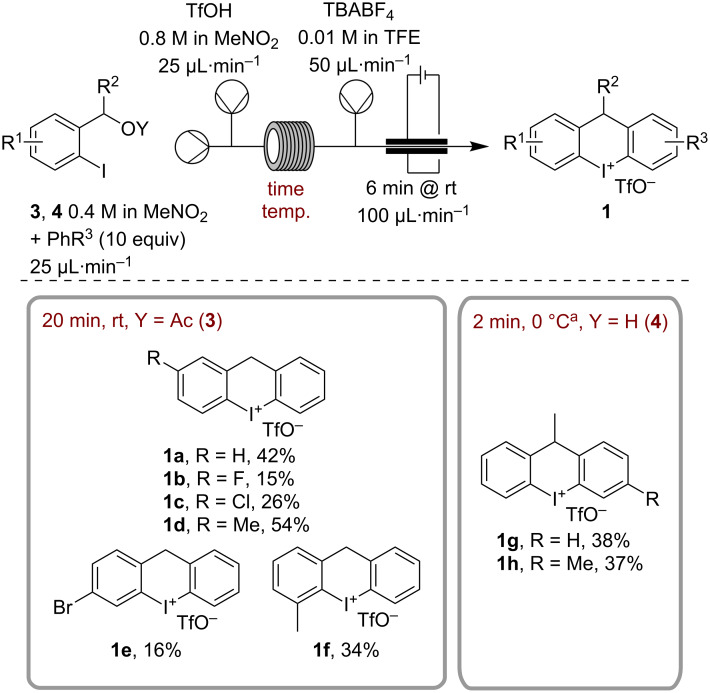
Substrate scope using the optimized conditions of Table 3. Yield is based on collecting for 3 h 20 min (2.00 mmol) after two reactor volumes had passed at the respective conditions. ^a^Solutions of benzyl alocohol and TfOH were precooled in flow before mixing (see Supporting Information File 1).

Finally, we investigated a variety of substituted benzyl acetates **3** (Figure 1). Substitutions *para* to the iodine led with F- and Cl-derivatives **1b** and **1c** to strongly diminished yields of 15% and 26%, respectively, due to expected lower yields in the Friedel–Crafts step. It was impossible to improve this significantly by increasing the time or temperature. In case of the more electron-rich derivative **1d**, we increased the yield to 54%. Other electron-withdrawing substituents show the same trend as in the Br-substituted **1e** yielding only 16%. Methylated substrate **1f** was isolated in 34% yield indicating that steric effects play a role during the oxidation step. We further wanted to investigate different arenes, but due to the lack of selectivity of the primary benzyl acetates in the Friedel–Crafts step, we were limited to *para*-substituted arenes, which unfortunately all resulted in the formation of insoluble intermediary iodoarenes. Derivatizing the benzylic position was done by employing secondary benzyl alcohols. These are well soluble and lead to an about 10-times shortened Friedel–Crafts step at 0 °C for the conversion of **3g**. Longer times only resulted in the decomposition of the intermediary iodoarene. Here it was possible to perform the procedure with either benzene or toluene, leading to a moderate yield of 38% and 37%. We could not use derivatives with any other substituents with secondary benzyl acetates since those led only to inseparable product mixtures.

## Conclusion

In summary, we have developed the first multi-step continuous-flow procedure for the generation of cyclic six-membered diaryliodonium salts. Starting from easily accessible benzyl acetates we were able to combine a Friedel–Crafts alkylation with a subsequent anodic oxidative cyclization in flow. The method is currently limited by the narrow starting materials being used due to the rather harsh conditions of those reactions. By addressing this issue, other substrates and higher yields could be realized in future.

## Supporting Information

File 1 Experimental, analytical data and copies of NMR spectra.
