# Supplementary material for: Two-step continuous-flow synthesis of 6-membered cyclic iodonium salts via anodic oxidation
Source: Beilstein J Org Chem. 2023 Jan 3;19:27–32. doi: 10.3762/bjoc.19.2 (PMC9830492; doi:10.3762/bjoc.19.2)
Supplement: File 1 — Experimental, analytical data and copies of NMR spectra. [file Beilstein_J_Org_Chem-19-27-s001.pdf]

BEILSTEIN JOURNAL OF ORGANIC CHEMISTRY

Supporting Information

for

Two-step continuous-flow synthesis of 6-membered cyclic iodonium salts via anodic oxidation

Julian Spils, Thomas Wirth and Boris J. Nachtsheim

Beilstein J. Org. Chem. **2023,** *19,* 27–32. doi:10.3762/bjoc.19.2

Experimental, analytical data and copies of NMR spectra

License and Terms: This is a supporting information file under the terms of the Creative Commons Attribution License (https://creativecommons.org/licenses/by/4.0). Please note that the reuse, redistribution and reproduction in particular requires that the author(s) and source are credited and that individual graphics may be subject to special legal provisions.
The license is subject to the *Beilstein Journal of Organic Chemistry* terms and conditions: (https://www.beilstein-journals.org/bjoc/terms)

Table of Contents

1 General Information S1

2 Optimization of Reaction Conditions S2

2.1.1 Optimization of the Oxidation and Cyclization in Batch S2

2.1.2 Optimization of the Oxidation and Cyclization in Flow S3

2.1.3 Final optimization of the one-pot-procedure in flow S4

3 Preparation of Starting Materials S5

3.1 General Procedures for the Synthesis of the Starting Materials S5

3.1.1 Sandmeyer-type Iodination of Anthranilic acids (GP1) S5

3.1.2 Borane-mediated Reduction of Carboxylic acids (GP2) S5

3.1.3 Iodine catalysed Acetylation of Benzylic alcohols (GP3) S6

3.2 Synthesis of Starting Materials S6

3.2.1 Primary Benzyl Acetates S6

3.2.2 Secondary Benzyl Alcohols S10

4 Substrate synthesis S12

4.1 General Procedures for the One-Pot Reaction Utilizing Primary Benzyl Acetates **3** and Secondary Benzyl Alcohol **4** (OPP1) S12

4.1.1 Standard conditions employing primary benzyl acetates **3** (OPP1a) S12

4.1.2 Standard conditions employing secondary benzyl alcohol **4** (OPP1b) S12

4.2 Substrates from Primary Benzyl Acetates and Secondary Benzyl Alcohols S13

4.3 Substrates that could not be successfully synthesized S16

5 NMR-Spectra of New Substrates S17

6 Literature S21

1 General Information

Unless otherwise noted, all reactions were carried out under air. Reactions with chemicals sensitive to moisture or oxygen were carried out under a nitrogen atmosphere using standard Schlenk techniques. All chemicals were purchased from commercial suppliers and either used as received or purified according to "Purification of Laboratory Chemicals".[1] Anhydrous tetrahydrofuran (THF) and diethyl ether (Et_2O) were obtained from an Inert PS-MD-6 solvent purification system. All other solvents were dried using standard methods if necessary.[1]

Yields refer to isolated yields of compounds estimated to be >95% pure as determined by ^{1}H-NMR spectroscopy.

Thin layer chromatography was performed on fluorescence indicator marked precoated silica gel 60 plates (Macherey-Nagel, ALUGRAM Xtra SIL G/UV254) and visualized by UV light (254 nm/366 nm). Flash column chromatography was performed on silica gel (0.040–0.063 mm) with the solvents given in the procedures.

NMR spectra were recorded on a Bruker Avance 360WB spectrometer, a Bruker Avance Neo 600 MHz spectrometer with BBO probe head and a Bruker Avance Neo 600 MHz spectrometer with TXI probe head at 23 °C. Chemical shifts for ^{1}H-NMR spectra are reported as δ (parts per million) relative to the residual proton signal of $CDCl_3$ at 7.26 ppm (s), DMSO-d_6 at 2.50 ppm (quint). Chemical shifts for ^{13}C-NMR spectra are reported as δ (parts per million) relative to the signal of $CDCl_3$ at 77.0 ppm (t), DMSO-d_6 at 39.5 ppm (sept). Chemical shifts for ^{19}F-NMR spectra are reported as δ (parts per million) relative to the signal of $Si(CH_3)_4$ at 0.00 ppm. The following abbreviations are used to describe splitting patterns: br. = broad, s = singlet, d = doublet, t = triplet, q = quartet, quint = quintet, sept = septet, m = multiplet. Coupling constants *J* are given in Hz.

APCI mass spectra were recorded on an Advion Expression CMS^L via ASAP probe or direct inlet. Low Resolution ESI mass spectra were recorded on an Agilent 6120 Series LC/MSD system. Low resolution EI mass spectra were recorded on an Agilent 5977A Series GC/MSD system. High resolution (HR) EI mass spectra were recorded on a double focusing mass spectrometer ThermoQuest MAT 95 XL from Finnigan MAT. HR-ESI and HR-APCI mass spectra were recorded on a Bruker Impact II. All Signals are reported with the quotient from mass to charge *m*/*z*.

IR spectra were recorded on a Nicolet Thermo iS10 scientific spectrometer with a diamond ATR unit. The absorption bands $\tilde{\nu}$ are reported in cm^{-1}.

Melting points of solids were measured on a Büchi M-5600 Melting Point apparatus and are uncorrected. The measurements were performed with a heating rate of 2 °C/min and the melting point temperatures *T* are reported in °C.

The electrochemical reactions in batch were carried out using a IKA Electrasyn 2.0 in a 5 mL glass vial with GC and Pt electrodes. For electrochemical reactions in flow in a galvanostatic mode a PEAKTECH 6225 A galvanostat was used. Electrolysis experiments were carried out using a Vapourtec Ion Electrochemical Reactor. Electrode materials: Platinum (Pt, coated obtained from Vapourtec Ltd.), Glassy Carbon (GC, SIGRADUR® from HTW Hochtemperatur-Werkstoffe GmbH). The electrodes (5 × 5 cm^2) are separated by a 0.5 mm spacer with a channel volume of 0.6 mL and an exposed electrode surface area of 12 cm^2 (each electrode). The syringe pumps that were used were Landgraf Laborsysteme HLL GmbH LA-30 syringe pumps.

2 Optimization of Reaction Conditions

2.1.1 Optimization of the Oxidation and Cyclization in Batch

*Table S1: Optimization of the Oxidation and Cyclization in Batch towards 10H-dibenzo[b,e]iodinin-5-ium salt (**1a**).*

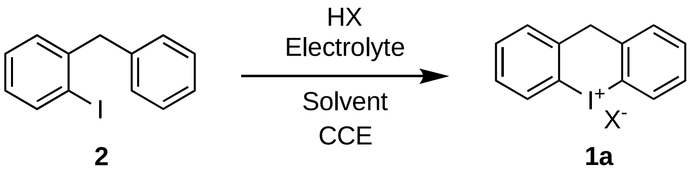

#[a]	HX (equiv)	Electrolyte (mM)	Solv. (mM)	Anode/ Cathode	Current (mA)	X	Yield / %
1	H_2SO_4 (2 M)		MeCN/Ac_2O 3:1 (40)	Pt/Pt	30 (2.4 F)	I	---
2	H_2SO_4 (2 M) **TfOH (5)**		MeCN/Ac_2O 3:1 (40)	Pt/Pt	**10** (2.4 F)	TfO	19
3	TfOH (5)	**Bu_4NBF_4 (5)**	**TFE (40)**	**GC**/Pt	10 (2.1 F)	TfO	45
4	TfOH (5)	Bu_4NBF_4 (5)	**MeCN**/TFE 4:1 (40)	GC/Pt	10 (2.0 F)	TfO	40
5	TfOH (5)	Bu_4NBF_4 (5)	**CH_2Cl_2**/TFE 4:1 (40)	GC/Pt	10 (2.2 F)	TfO	46
6	**TfOH (15)**	Bu_4NBF_4 (5)	CH_2Cl_2/TFE 4:1 (40)	GC/Pt	10 (3.0 F)	TfO	44
7	TfOH (5)	**Bu_4NBF_4 (15)**	CH_2Cl_2/TFE 4:1 (40)	GC/Pt	10 (2.2 F)	TfO	44
8	TfOH (5)	Bu_4NBF_4 (5)	CH_2Cl_2/**HFIP** 4:1 (40)	GC/Pt	10 (2.0 F)	TfO	78
9	**TfOH (2)**	Bu_4NBF_4 (5)	CH_2Cl_2/HFIP 4:1 (40)	GC/Pt	10 (2.0 F)	TfO	76
10	TfOH (2)	Bu_4NBF_4 (5)	CH_2Cl_2/HFIP 4:1 (40)	GC/Pt	**30** (2.3 F)	TfO	57

[a] All reactions were performed on a 0.200 mmol scale with an IKA Electrasyn 2.0 in an undivided cell.

2.1.2 Optimization of the Oxidation and Cyclization in Flow

Table S2: Optimization of the Oxidation and Cyclization in Flow towards 10H-dibenzo[b,e]iodinin-5-ium salt (**1a**).

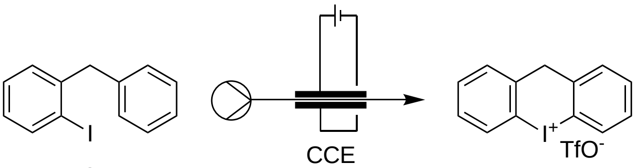

#[a]	HX (equiv)	Additives	Solv. (mM)	Anode/ Cathode	Current (mA)	Yield /%
7	TfOH (1)	Bu_4NBF_4 (5 mM)	CH_2Cl_2/TFE 1:1 (100)	GC/Pt	32 (2.0 F)	62
8	TfOH (2)	Bu_4NBF_4 (5 mM)	CH_2Cl_2/TFE 1:1 (100)	GC/Pt	32 (2.0 F)	74
9[b]	**HBF_4OEt_2 (1)**	Bu_4NBF_4 (5 mM)	CH_2Cl_2/TFE 1:1 (100)	GC/Pt	32 (2.0 F)	66
10[b]	HBF_4OEt_2 (1)	Bu_4NBF_4 (5 mM)	CH_2Cl_2/TFE 1:1 (100)	GC/Pt	**48 (3.0 F)**	62
11	TfOH (2)	Bu_4NBF_4 (5 mM) **PhH (9 equiv)**	**$MeNO_2$ (100)**	GC/Pt	32 (2.0 F)	48
13	TfOH (2)	Bu_4NBF_4 (5 mM) PhH (9 equiv)	**$MeNO_2$/TFE 1:1** (100)	GC/Pt	32 (2.0 F)	63
14	TfOH (2)	**PhH (9 equiv)**	$MeNO_2$/TFE 1:1 (100)	GC/Pt	32 (2.0 F)	62
15	TfOH (2)	PhH (9 equiv)	$MeNO_2$/TFE 1:1 (100)	GC/Pt	**48 (3.0 F)**	62

[a] Reaction was performed in a Vapourtec Ion electrochemical flow reactor with a glassy carbon (GC) anode, a Pt cathode and a 0.5 mm PTFE spacer in between. General reaction conditions: **1** (0.1 M), $TBABF_4$ (0.005 M). Flowrate: 0.1 ml min^{-1}. Yield is based on collecting for 20 min (0.200 mmol) after two reactor volumes had passed at the respective condition. [b] The corresponding tetrafluoroborate was isolated.

2.1.3 Final optimization of the one-pot-procedure in flow

*Table S1: Final optimization of the one-pot-procedure in flow towards 10H-dibenzo[b,e]iodinin-5-ium salt (**1a**).*

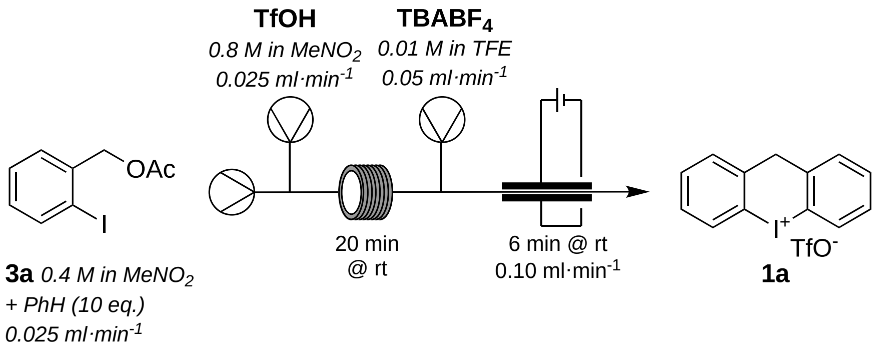

#[a]	Electrolyte	Current (mA)	Yield /%
1	Bu_4NBF_4	32 (2.0 F)	54
2	Bu_4NBF_4	**48 (3.0 F)**	54
3	---	32 (2.0 F)	49
4[b]	---	32 (2.0 F)	38 (46, 37, 34, 33)
5[b]	**Bu_4NBF_4**	32 (2.0 F)	43 (52, 41, 41, 38)

[a] Reaction was performed in a Vapourtec Ion electrochemical flow reactor with a glassy carbon (GC) anode, a Pt cathode and a 0.5 mm PTFE spacer in between. Yield is based on collecting for 20 min (0.200 mmol) after two reactor volumes had passed at the respective condition.

[b] **1a** was collected for 3 h 20 min (2.00 mmol) divided in four fractions each 50 min (0.500 mmol). Yields of each fraction is in brackets.

3 Preparation of Starting Materials

3.1 General Procedures for the Synthesis of the Starting Materials

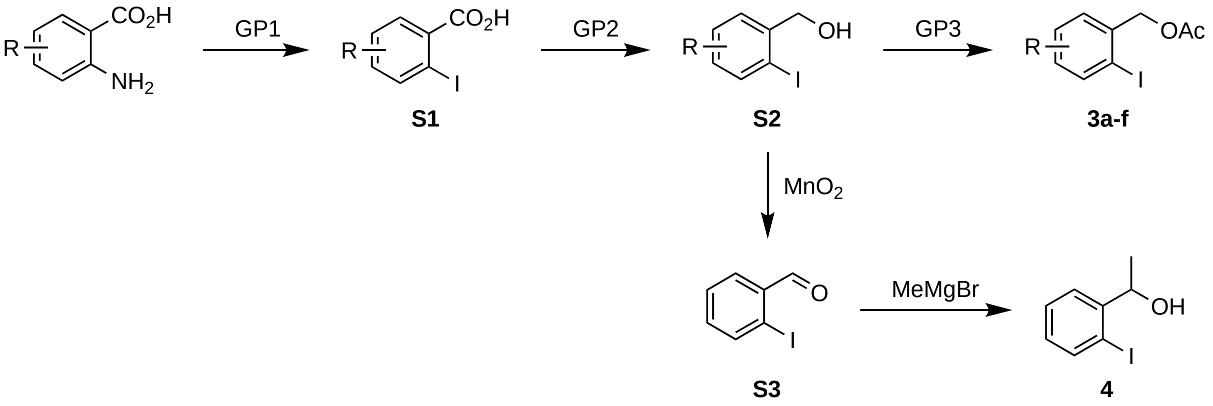

*Scheme S1: General scheme for the synthesis of primary benzyl acetates **3** and secondary benzyl alcohol **4**.*

3.1.1 Sandmeyer-type Iodination of Anthranilic acids (GP1)

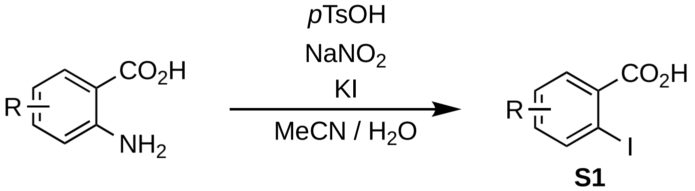

Following a reported procedure,[1] the anthranilic acid derivative (1.00 equiv) and $TsOH\cdot H_2O$ (3.00 equiv) were suspended in MeCN (0.125 M). The mixture was cooled to 10–15 °C. A solution of $NaNO_2$ (2.00 equiv) and KI (2.50 equiv) in H_2O (1.5 ml/mmol) was added dropwise over 1 h. After complete addition, the resulting dark coloured mixture was stirred for another 1 h and then 1 N HCl (5 ml/mmol) and EtOAc (10 ml/mmol) were added. The phases were separated and the aqueous phase was extracted with EtOAc (2 × 5 ml/mmol). The combined organic phases were washed with water (5 ml/mmol), $Na_2S_2O_3$ (10% *w*/*w*, 5 ml/mmol) and brine (2 ml/mmol), dried over Na_2SO_4 and concentrated under reduced pressure. The residue was either purified by filtration over silica or by column chromatography.

3.1.2 Borane-mediated Reduction of Carboxylic acids (GP2)

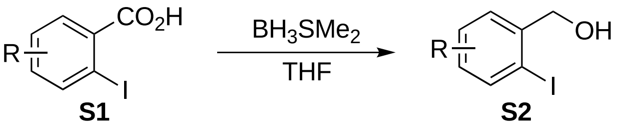

In modification of a reported procedure,[2] the *o*-iodobenzoic acid derivative (**S1**, 1.00 equiv) was dissolved in dry THF (1.00 M). After cooling to 0 °C, BH_3SMe_2 (1.20 equiv) were added dropwise over 15 min. After the addition, the mixture was allowed to warm to rt and stirred for 16 h. After completion of the reaction, the mixture was cooled to 0 °C and MeOH (40 µl/mmol) was carefully added. Afterwards, 1 M Na_2CO_3 solution (0.5 ml/mmol) was slowly added. The resulting mixture was diluted with water (3 ml/mmol) and extracted with Et_2O (3 × 5 ml/mmol). The combined organic phases were washed with brine (5 ml/mmol), dried over Na_2SO_4 and concentrated under reduced. The crude product was purified by column chromatography on silica gel.

3.1.3 Iodine catalysed Acetylation of Benzylic alcohols (GP3)

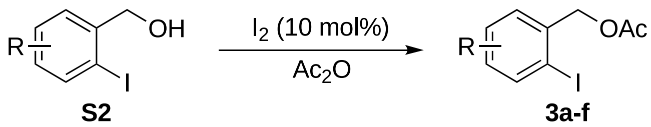

In modification of a reported procedure,[3] the *o*-iodobenzyl alcohol derivative (**S2**, 1.00 equiv) was suspended in Ac_2O (6.00 equiv). If necessary, dichloromethane (0.1–0.2 ml/mmol) was added. Iodine (0.100 equiv) was added and the mixture was stirred at rt. The conversion was monitored via TLC and after full conversion the mixture was diluted with water (0.5 ml/mmol) and sat. $NaHCO_3$ solution (2 ml/mmol). The aqueous phase was extracted with dichloromethane (1 ml/mmol). The combined organic phases were washed with sat. $Na_2S_2O_3$ solution, dried over Na_2SO_4 and concentrated under reduced pressure. The crude product was either used as received or purified by column chromatography on silica gel.

3.2 Synthesis of Starting Materials

3.2.1 Primary Benzyl Acetates

The *o*-iodobenzoic acid derivative **S1a** were commercially available and have been used as received for the synthesis of the corresponding alcohols.

2-Iodobenzyl acetate (3a)

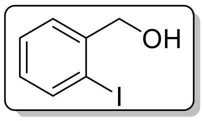

Following a reported procedure,[1] *o*-iodobenzoic acid (49.6 g, 200 mmol 1.00 equiv) was dissolved in dry THF (200 ml). After cooling to 0 °C, $NaBH_4$ (21.9 g, 580 mmol, 2.90 equiv) was added portionwise. To the resulting mixture, I_2 (38.1 g, 150 mmol, 0.750 equiv) in THF (300 ml) was slowly added over 5 h. After the addition, the mixture was allowed to warm to rt and stirred overnight. After completion of the reaction, the mixture was cooled to 0 °C and water (70 ml) was carefully added. Afterwards, 3 M HCl was slowly added until the solution was at pH 2. The resulting mixture was extracted with Et_2O (4 × 150 ml). The combined organic phases were washed with 1 M Na_2CO_3 (200 ml), sat. $Na_2S_2O_3$ (50 ml) and brine (200 ml), dried over Na_2SO_4, and concentrated under reduced pressure. Recrystallization of the crude product from cyclohexane gave (2-iodophenyl)methanol (**S2a**, 46.8 g, 200 mmol, quant.) as a white crystalline solid.

^{1}H-NMR (601 MHz, $CDCl_3$) δ = 7.83 (dd, *J* = 7.8, 1.2 Hz, 1H), 7.46 (dd, *J* = 7.6, 1.7 Hz, 1H), 7.37 (td, *J* = 7.5, 1.2 Hz, 1H), 7.00 (td, *J* = 7.6, 1.8 Hz, 1H), 4.68 (d, *J* = 6.3 Hz, 2H), 2.00 (t, *J* = 6.3 Hz, 1H). **^{13}C-NMR (151 MHz, CDCl3)** δ = 142.9, 139.5, 129.6, 128.8, 128.7, 97.7, 69.6. **FTIR (ATR, neat)** $\tilde{\nu}$ = 3262, 3061, 2887, 2843, 1434, 1321, 1195, 1033, 1009, 738. **MS (EI, 70 eV)** *m/z* = 234.0 $[M]^{+\cdot}$ **Mp** *T* = 91. The analytical data is in accordance with literature data.[1]

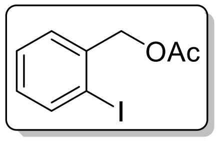

Following **GP3**, the reaction of (2-iodophenyl)methanol (**S2a**, 4.68 g, 20.0 mmol) in Ac_2O (11.3 ml, 120 mmol) with iodine (508 mg, 2.00 mmol) gave after a reaction time of 4 h and subsequent column chromatography (silica, cyclohexane) 2-Iodobenzyl acetate (**3a**, 5.53 g, 20.0 mmol, quant.) as a colourless liquid.

^{1}H-NMR (600 MHz, $CDCl_3$) δ = 7.86 (d, *J* = 7.9 Hz, 1H), 7.38 (dd, *J* = 7.6, 1.9 Hz, 1H), 7.35 (td, *J* = 7.4, 0.9 Hz, 1H), 7.03 (td, *J* = 7.6, 1.9 Hz, 1H), 5.13 (s, 2H), 2.15 (s, 3H). **^{13}C-NMR (151 MHz, $CDCl_3$)** δ = 170.6, 139.5, 138.3, 129.8, 129.5, 128.3, 98.4, 70.1, 20.9. **FTIR (ATR, neat)** $\tilde{\nu}$ = 3056, 2953, 1732, 1566, 1437,

1378, 1360, 1218, 1012, 746. **MS (EI, 70 eV)** *m/z* = 276.0 $[M]^{+\cdot}$, 149.1 $[M-I]^{+}$. The analytical data is in accordance with literature data.[1]

5-Fluoro-2-iodobenzyl acetate (3b)

Following **GP1**, the reaction of 2-amino-5-fluorobenzoic acid (7.76 g, 50.0 mmol) and TsOH·H_2O (28.5 g, 150 mmol) in MeCN (400 ml) with $NaNO_2$ (6.90 g, 100 mmol) and KI (20.8 g, 125 mmol) in water (75 ml) gave 5-fluoro-2-iodobenzoic acid (**S1b**, 11.4 g, 43.0 mmol, 86%) as a yellow solid.

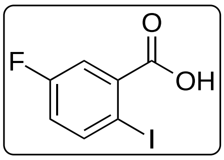

^{1}H-NMR (600 MHz, DMSO-*d$_6$*) δ = 8.31 (s, 1H), 7.88 (dd, *J* = 8.5, 2.6 Hz, 1H), 7.82 (dd, *J* = 8.7, 6.0 Hz, 1H), 7.35 (td, *J* = 8.4, 2.6 Hz, 1H). **^{13}C-NMR (151 MHz, DMSO-*d$_6$*)** δ = 167.1, 162.5 (d, *J* = 254.2 Hz), 132.9 (d, *J* = 2.4 Hz), 132.2 (d, *J* = 9.0 Hz), 127.5 (d, *J* = 23.7 Hz), 115.2 (d, *J* = 21.3 Hz), 95.3 (d, *J* = 8.5 Hz). **^{19}F-NMR (565 MHz, $CDCl_3$)** δ = -108.3 (m). **FTIR (ATR, neat)** $\tilde{\nu}$ = 2977, 2856, 2648, 1694, 1573, 1411, 1300, 1257, 1203, 768. **MS (EI, 70 eV)** *m/z* = 265.9 $[M]^{+\cdot}$. **Mp** *T* = 135 – 137. The analytical data is in accordance with literature data.[4]

Following **GP2**, the reaction of 5-fluoro-2-iodobenzoic acid (**S1b**, 10.6 g, 40.0 mmol) in dry THF (40 ml) with BH_3SMe_2 (4.80 ml, 48.0 mmol) gave (5-fluoro-2-iodophenyl)methanol (**S2b**, 9.56 g, 37.9 mmol, 95%) as a colourless solid.

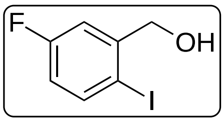

^{1}H-NMR (601 MHz, $CDCl_3$) δ = 7.74 (dd, *J* = 8.6, 3.1 Hz, 1H), 7.25 (dd, *J* = 9.3, 3.1 Hz, 1H), 6.76 (td, *J* = 8.3, 3.1 Hz, 1H), 4.63 (d, *J* = 6.1 Hz, 2H), 2.07 (t, *J* = 6.1 Hz, 1H). **^{13}C-NMR (151 MHz, $CDCl_3$)** δ = 163.1 (d, *J* = 247.8 Hz), 145.0 (d, *J* = 6.9 Hz), 140.1 (d, *J* = 7.6 Hz), 116.4 (d, *J* = 22.0 Hz), 115.5 (d, *J* = 23.4 Hz), 89.1 (d, *J* = 2.7 Hz), 68.8. **^{19}F-NMR (565 MHz, $CDCl_3$)** δ = −113.4. **FTIR (ATR, neat)** $\tilde{\nu}$ = 3286, 1578, 1458, 1437, 1356, 1263, 1149, 1058, 1013, 808. **MS (EI, 70 eV)** *m/z* = 251.9 $[M]^{+\cdot}$. **Mp** *T* = 111.5 – 113. The analytical data is in accordance with literature data.[1]

Following **GP3**, the reaction of (5-fluoro-2-iodophenyl)methanol (**S2b**, 1.76 g, 7.00 mmol) in Ac_2O (4.00 ml, 42.0 mmol) with iodine (178 mg, 0.700 mmol) gave after a reaction time of 4 h and subsequent column chromatography (silica, cyclohexane/ethyl acetate 1:0→19:1) 5-fluoro-2-iodobenzyl acetate (**3b**, 1.80 g, 6.12 mmol, 87%) as a colourless solid.

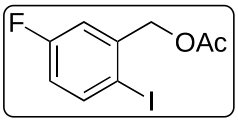

^{1}H-NMR (600 MHz, $CDCl_3$) δ = 7.59 (dd, *J* = 8.0, 2.6 Hz, 1H), 7.36 (dd, *J* = 8.6, 5.8 Hz, 1H), 7.08 (td, *J* = 8.3, 2.6 Hz, 1H), 5.10 (s, 2H), 2.13 (s, 3H). **^{13}C-NMR (151 MHz, $CDCl_3$)** δ = 170.6, 161.7 (d, *J* = 252.4 Hz), 134.4 (d, *J* = 3.5 Hz), 130.6 (d, *J* = 8.3 Hz), 126.5 (d, *J* = 23.8 Hz), 115.4 (d, *J* = 20.9 Hz), 97.9 (d, *J* = 8.3 Hz), 69.3, 20.9. **^{19}F-NMR (565 MHz, $CDCl_3$)** δ = -112.1. **FTIR (ATR, neat)** $\tilde{\nu}$ = 2928, 1731, 1716, 1584, 1480, 1438, 1359, 1237, 1220, 1048, 1026, 868. **HRMS (ESI+, MeOH)** *m/z* = 316.94410 $[M+Na]^{+}$. Calculated for $[C_9H_8FINaO_2]^{+}$: *m/z* = 316.94453. **Mp** *T* = 38 – 39.

5-Chloro-2-iodobenzyl acetate (3c)

Following **GP1**, the reaction of 2-amino-5-chlorobenzoic acid (5.15 g, 30.0 mmol) and TsOH·H_2O (17.1 g, 90.0 mmol) in MeCN (240 ml) with $NaNO_2$ (4.14 g, 60.0 mmol) and KI (12.5 g, 75.0 mmol) in water (45 ml) gave 5-chloro-2-iodobenzoic acid (**S1c**, 8.21 g, 29.1 mmol, 97%) as a yellow solid.

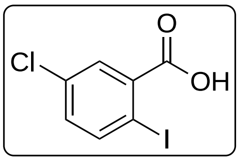

^{1}H-NMR (600 MHz, DMSO-*d$_6$*) δ = 13.61 (brs, 1H), 7.97 (d, *J* = 8.4 Hz, 1H), 7.72 (d, *J* = 2.6 Hz, 1H), 7.32 (dd, *J* = 8.4, 2.6 Hz, 1H). **^{13}C-NMR (151 MHz, DMSO-*d$_6$*)** δ = 167.0, 142.1, 138.9, 133.2, 132.2, 129.5,

92.1. **FTIR (ATR, neat)** $\tilde{\nu}$ = 1694, 1673, 1574, 1400, 1290, 1246, 1117, 1017, 780, 749. **MS (EI, 70 eV)** *m/z*: 281.9 $[M]^{+\cdot}$. **Mp** *T* = 154 – 157. The analytical data is in accordance with literature data.[1]

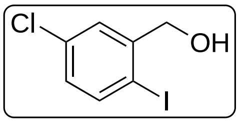

Following **GP2**, the reaction of 5-chloro-2-iodobenzoic acid (**S1c**, 20.0 mmol, 5.65 g) in dry THF (20 ml) with BH_3SMe_2 (24.0 mmol, 2.40 ml) gave after a subsequent column chromatography (silica, cyclohexane/ethyl acetate 1:0→9:1) (5-chloro-2-iodophenyl)methanol (**S2c**, 4.15 g, 15.5 mmol, 77%) as a colourless solid.

^{1}H-NMR (601 MHz, CDCl$_3$) δ = 7.71 (d, *J* = 8.3 Hz, 1H), 7.48 (d, *J* = 2.5 Hz, 1H), 6.99 (dd, *J* = 8.3, 2.6 Hz, 1H), 4.63 (s, 2H), 2.10 (s, 1H). **^{13}C-NMR (151 MHz, CDCl$_3$)** δ = 144.3, 140.0, 135.0, 129.2, 128.2, 93.6, 68.7. **FTIR (ATR, neat)** $\tilde{\nu}$ = 3289, 3212, 1452, 1436, 1391, 1193, 1101, 1055, 1007, 974, 808. **MS (EI, 70 eV)** *m/z*: 268.0 $[M]^{+\cdot}$, 233.9 $[M - Cl]^+$, 141.0 $[M - I]^+$. **Mp** *T* = 118 – 119.5. The analytical data is in accordance with literature data.[1]

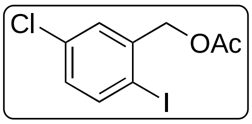

Following **GP3**, the reaction of (5-chloro-2-iodophenyl)methanol (**S2c**, 4.40 mmol, 1.18 g) in Ac_2O (26.4 mmol, 2.50 ml) with iodine (0.440 mmol, 112 mg) gave after a reaction time of 4 h and subsequent column chromatography (silica, cyclohexane/ethyl acetate 1:0→19:1) 5-chloro-2-iodobenzyl acetate (**3c**, 1.28 g, 4.12 mmol, 94%) as a colourless solid.

^{1}H-NMR (600 MHz, CDCl$_3$) δ = 7.76 (d, *J* = 8.4 Hz, 1H), 7.36 (d, *J* = 2.5 Hz, 1H), 7.02 (dd, *J* = 8.4, 2.6 Hz, 1H), 5.07 (s, 2H), 2.17 (s, 3H). **^{13}C-NMR (151 MHz, CDCl$_3$)** δ = 170.4, 140.4, 140.1, 134.8, 129.8, 129.1, 94.6, 69.4, 20.9. **FTIR (ATR, neat)** $\tilde{\nu}$ = 3054, 1730, 1437, 1375, 1360, 1248, 1240, 1094, 1047, 874, 819. **HRMS (ESI+, MeOH)** *m/z* = 332.91460 $[M+Na]^+$. Calculated for $[C_9H_8ClINaO_2]^+$: *m/z* = 332.91498. **Mp** *T* = 80 – 82.

2-Iodo-5-methyl-benzyl acetate (3d)

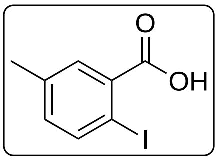

Following a modified literature procedure,[2] 2-amino-5-methylbenzoic acid (3.02 g, 20.0 mmol) was suspended in conc. HCl (15 ml) and was stirred for 15 min at 0 °C before $NaNO_2$ (1.79 g, 26.0 mmol) in H_2O (4 ml) was added dropwise over 10 min. Stirring continued for 1.5 h at 0 °C and afterwards KI (16.6 g, 100 mmol) in H_2O (20 mL) was added dropwise over 20 min. The reaction mixture was allowed to warm to room temperature and was stirred for 24 h. H_2O (40 mL) was added and extracted with EtOAc (3 × 40 mL). The combined organic phases were washed with sat. $Na_2S_2O_3$-solution (20 ml), brine (40 ml) and H_2O (40 ml), dried over Na_2SO_4, filtered and concentrated *in vacuo* to give 2-iodo-5-methylbenzoic acid (**S1d**, 5.04 g, 20.0 mmol, 96%) as a yellow solid.

^{1}H-NMR (600 MHz, CDCl$_3$) δ = 7.91 (d, *J* = 8.1 Hz, 1H), 7.84 (d, *J* = 2.2 Hz, 1H), 7.02 (dd, *J* = 8.2, 2.2 Hz, 1H), 2.35 (s, 3H). **^{13}C-NMR (151 MHz, CDCl$_3$)** δ = 13C NMR (151 MHz, CDCl3) δ = 171.7, 141.7, 138.2, 134.6, 132.8, 90.6, 20.8. **FTIR (ATR, neat)** $\tilde{\nu}$ = 2870, 2549, 1674, 1560, 1468, 1408, 1296, 1255, 1215, 1012, 756. **MS (EI, 70 eV)** *m/z* = 261.9 $[M]^{+\cdot}$. **Mp** *T* = 111 – 113. The analytical data is in accordance with literature data.[5]

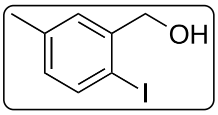

Following **GP2**, the reaction of 2-iodo-5-methylbenzoic acid (**S1d**, 15.0 mmol, 3.93 g) in dry THF (15 ml) with BH_3SMe_2 (18.0 mmol, 1.80 ml) gave (2-iodo-5-methylphenyl)methanol (**S2d**, 3.72 g, 15.0 mmol, quant.) as a colourless solid, which was analysed via ^{1}H-NMR and used without further purification.

¹H-NMR (600 MHz, CDCl₃) δ = 7.68 (d, J = 8.0 Hz, 1H), 7.28 (d, J = 2.2 Hz, 1H), 6.83 (dd, J = 8.0, 2.2 Hz, 1H), 4.65 (s, 2H), 3.77 – 3.73 (m, 1H), 2.32 (s, 3H). The analytical data is in accordance with literature data.[6]

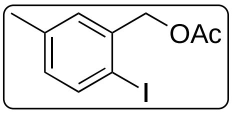

Following **GP3**, the reaction of (2-iodo-5-methylphenyl)methanol (**S2d**, 10.0 mmol, 2.48 g) in Ac_2O (60.0 mmol, 5.67 ml) with iodine (1.00 mmol, 254 mg) gave after a reaction time of 4 h and subsequent column chromatography (silica, cyclohexane/ethyl acetate 1:0→19:1) 2-iodo-3-methylbenzyl acetate (**3d**, 2.27 g, 7.82 mmol, 78%) as a yellow solid.

¹H-NMR (600 MHz, CDCl₃) δ = 7.72 (d, J = 8.0 Hz, 1H), 7.19 (d, J = 2.2 Hz, 1H), 6.85 (dd, J = 8.0, 2.2 Hz, 1H), 5.09 (s, 2H), 2.31 (s, 3H), 2.14 (s, 3H). **¹³C-NMR (151 MHz, CDCl₃)** δ = 170.6, 139.3, 138.5, 137.9, 130.9, 130.5, 129.0, 94.4, 70.1, 20.9. **FTIR (ATR, neat)** $\tilde{\nu}$ = 2974, 2916, 1727, 1443, 1375, 1357, 1233, 1045, 1020, 812. **HRMS (ESI+, MeOH)** m/z = 312.96956 [M+Na]⁺. Calculated for $[C_{10}H_{11}INaO_2]^+$: m/z = 312.96960. **Mp** T = 103 – 104.

4-Bromo-2-iodobenzyl acetate (3e)

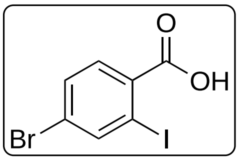

Following **GP1**, the reaction of 2-amino-4-bromobenzoic acid (10.0 mmol, 2.16 g) in MeCN (80 ml) with $NaNO_2$ (1.38 g, 20.0 mmol) and KI (4.15 g, 25.0 mmol) in water (15 ml) gave 4-bromo-2-iodobenzoic acid (**S1e**, 2.65 g, 8.11 mmol, 81%) as a colourless solid.

¹H-NMR (600 MHz, DMSO-*d*₆) δ = 13.47 (s, 1H), 8.19 (d, J = 1.8 Hz, 1H), 7.70 (dd, J = 8.3, 1.8 Hz, 1H), 7.65 (d, J = 8.3 Hz, 1H). **¹³C-NMR (151 MHz, DMSO- *d*₆)** δ = 167.4, 142.1, 136.0, 131.5, 131.2, 125.0, 95.7. **FTIR (ATR, neat)** $\tilde{\nu}$ = 3064, 1672, 1422, 1291, 1542, 1291, 1249, 1023, 838, 771. **MS (EI, 70 eV)** m/z = 325.8 $[M]^{+\cdot}$. **Mp** T = 175 – 177. The analytical data is in accordance with literature data.[1]

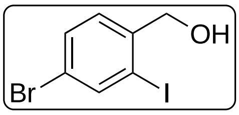

Following **GP2**, the reaction of 4-bromo-2-iodobenzoic acid (**S1e**, 7.50 mmol, 2.45 g) in dry THF (7.5 ml) with BH_3SMe_2 (9.00 mmol, 900 µl) gave after a subsequent column chromatography (silica, cyclohexane/ethyl acetate 1:0→9:1) (2-iodo-4-bromophenyl)methanol (**S2e**, 1.63 g, 5.21 mmol, 69%) as a colourless solid.

¹H-NMR (600 MHz, CDCl₃) δ = 7.96 (d, J = 1.9 Hz, 1H), 7.50 (dd, J = 8.2, 1.9 Hz, 1H), 7.33 (d, J = 8.2 Hz, 1H), 4.62 (d, J = 5.6 Hz, 2H), 2.06 (t, J = 6.0 Hz, 1H). **¹³C-NMR (151 MHz, CDCl₃)** δ = 141.6, 140.9, 131.5, 129.3, 121.7, 97.3, 68.6. **FTIR (ATR, neat)** $\tilde{\nu}$ = 3315, 3226, 1572, 1550, 1467, 1377, 1056, 1008, 801, 705. **MS (EI, 70 eV)** m/z = 311.8 $[M]^{+\cdot}$. **Mp** T = 104 – 105. The analytical data is in accordance with literature data.[1]

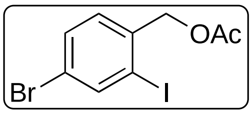

Following **GP3**, the reaction of (2-iodo-4-bromophenyl)methanol (**S2e**, 5.11 mmol, 1.60 g) in Ac_2O (30.7 mmol, 3.13 ml) with iodine (0.511 mmol, 130 mg) gave after a reaction time of 4 h and subsequent column chromatography (silica, cyclohexane/ethyl acetate 1:0 → 19:1) 5-chloro-2-iodobenzyl acetate (**3e**, 1.73 g, 4.87 mmol, 95%) as a colourless liquid.

¹H-NMR (600 MHz, CDCl₃) δ = 7.69 (d, J = 8.3 Hz, 1H), 7.50 (d, J = 2.3 Hz, 1H), 7.16 (dd, J = 8.4, 2.4 Hz, 1H), 5.07 (s, 2H), 2.17 (s, 3H). **¹³C-NMR (151 MHz, CDCl₃)** δ = 170.4, 140.7, 140.4, 132.8, 132.0, 122.7,

95.7, 69.3, 20.9. **FTIR (ATR, neat)** $\tilde{\nu}$ = 3079, 2934, 1734, 1574, 1552, 1462, 1367, 1216, 1020, 809. **HRMS (ESI+, MeOH)** *m/z* = 376.86414 [M+Na]$^+$. Calculated for $[C_9H_8BrINaO_2]^+$: *m/z* = 376.86446.

2-Iodo-3-methylbenzyl acetate (3f)

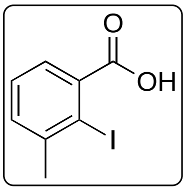

Following **GP1**, the reaction of 2-amino-3-methylbenzoic acid (30.0 mmol, 4.53 g) in MeCN (240 ml) with $NaNO_2$ (4.14 g, 60.0 mmol) and KI (12.5 g, 75.0 mmol) in water (45 ml) gave 2-iodo-3-methylbenzoic acid (**S1f**, 3.26 g, 12.4 mmol, 41%) as a yellow solid.

^{1}H-NMR (600 MHz, DMSO-*d$_6$*) δ = 13.07 (s, 1H), 7.42 (dd, *J* = 7.2, 2.1 Hz, 1H), 7.34 (t, *J* = 7.4 Hz, 1H), 7.30 (dd, *J* = 7.6, 2.2 Hz, 1H), 2.43 (s, 3H). **^{13}C-NMR (151 MHz, DMSO-*d$_6$*)** δ = 169.7, 142.4, 140.3, 131.2, 128.0, 126.1, 99.6, 29.0. **FTIR (ATR, neat)** $\tilde{\nu}$ = 2975, 2805, 2545, 1698, 1671, 1568, 1413, 1290, 1187, 929. **MS (EI, 70 eV)** *m/z*: 261.9 [M]$^{+\cdot}$. **Mp** *T* = 151 – 152. The analytical data is in accordance with literature data.[7]

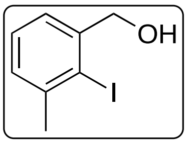

Following **GP2**, the reaction of 2-iodo-3-methylbenzoic acid (**S1f**, 11.3 mmol, 2.97 g) in dry THF (11 ml) with BH_3SMe_2 (13.6 mmol, 1.36 ml) gave after a subsequent column chromatography (silica, cyclohexane/ethyl acetate 1:0 → 9:1) (2-iodo-3-methylphenyl)methanol (**S2f**, 2.10 g, 8.47 mmol, 75%) as a colourless solid.

^{1}H-NMR (360 MHz, CDCl$_3$) δ = 7.28 – 7.23 (m, 2H), 7.25 – 7.14 (m, 1H), 4.71 (s, 2H), 2.48 (s, 3H). **^{13}C-NMR (91 MHz, CDCl$_3$)** δ = 143.2, 142.1, 129.0, 128.1, 125.7, 104.7, 70.3, 29.1. **FTIR (ATR, neat)** $\tilde{\nu}$ = 3251, 3070, 2891, 1573, 1445, 1375, 1312, 1169, 1055. 1000. **MS (EI, 70 eV)** *m/z*: 248.0 [M]$^{+\cdot}$. **Mp** *T* = 70 – 71. The analytical data is in accordance with literature data.[8]

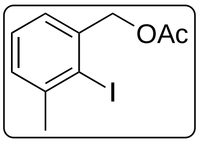

Following **GP3**, the reaction of (2-iodo-3-methylphenyl)methanol (**S2f**, 8.00 mmol, 1.98 g) in Ac_2O (48.0 mmol, 4.54 ml) with iodine (0.800 mmol, 203 mg) gave after a reaction time of 4 h and subsequent column chromatography (silica, cyclohexane/ethyl acetate 1:0 → 19:1) 2-iodo-3-methylbenzyl acetate (**3f**, 2.19 g, 7.57 mmol, 95%) as a colourless liquid.

^{1}H-NMR (600 MHz, CDCl$_3$) δ = 7.25 – 7.19 (m, 2H), 7.17 (dd, J = 7.2, 2.0 Hz, 1H), 5.17 (s, 2H), 2.49 (s, 3H), 2.15 (s, 3H). **^{13}C-NMR (151 MHz, CDCl$_3$)** δ = 170.7, 142.6, 138.8, 129.5, 128.0, 126.6, 105.5, 71.1, 29.3, 21.0. **FTIR (ATR, neat)** $\tilde{\nu}$ = 3049, 2948, 1734, 1532, 1450, 1372, 1218, 1054, 1010, 773. **HRMS (ESI+, MeOH)** *m/z* = 312.96922 [M+Na]$^+$. Calculated for $[C_{10}H_{11}INaO_2]^+$: *m/z* = 312.96960.

3.2.2 Secondary Benzyl Alcohols

1-(2-Iodophenyl)ethan-1-ol (4)

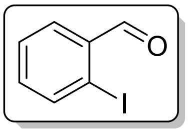

(2-Iodophenyl)methanol (**S2a**, 24.9 g, 106 mmol, 1.00 equiv) was dissolved in $CHCl_3$ (265 mL), activated MnO_2 (92.4 g, 1.06 mol, 10.0 equiv) was added and the suspension was stirred for 24 h at rt. The mixture was filtrated over Celite and the filter material was washed with ethyl acetate (300 mL). After removal of the solvent under reduced pressure, 2-iodobenzaldehyde (**S3**, 21.9 g, 94.4 mmol, 89%) was obtained as a colourless solid.

^{1}H-NMR (600 MHz, CDCl$_3$) δ = 10.07 (d, *J* = 0.7 Hz, 1H), 7.96 (dd, *J* = 7.9, 1.1 Hz, 1H), 7.89 (dd, *J* = 7.7, 1.8 Hz, 1H), 7.47 (t, *J* = 7.5 Hz, 1H), 7.29 (ddd, *J* = 7.9, 7.4, 1.8 Hz, 1H). **^{13}C-NMR (151 MHz, CDCl$_3$)** δ =

196.0, 140.8, 135.7, 135.2, 130.4, 128.9, 100.9. **FTIR (ATR, neat)** $\tilde{\nu}$ = 2849, 2833, 2746, 1683, 1578, 1436, 1388, 1261, 1200, 1015, 748. **MS (EI, 70 eV)** *m/z* = 232.0 $[M]^{+\cdot}$, 203.0 $[M\text{-}CHO]^{+}$. **Mp** *T* = 34 – 35 °C. The analytical data is in accordance with literature data.[1]

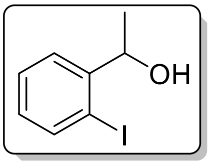

To a solution of 2-iodobenzaldehyde (**S3**, 3.02 g, 13.0 mmol, 1.00 equiv) in dry THF (26 mL) at 0 °C MeMgBr (4.80 mL, 3 M in THF, 14.3 mmol, 1.10 equiv) was added at a rate of 0.8 mL/min. The mixture was allowed to warm to rt overnight and sat. NH_4Cl (10 mL) and Et_2O (10 mL) were added. The phases were separated and the aqueous phase was extracted with Et_2O (2 × 10 mL). The combined organic phases were dried over Na_2SO_4, concentrated under reduced pressure and the residue was purified via column chromatography (silica, cyclohexane/ethyl acetate 1:0→10:1) to obtain 1-(2-iodophenyl)ethan-1-ol (**4**, 2.84 g, 8.38 mmol, 88%) as a colourless liquid.

^{1}H-NMR (600 MHz, $CDCl_3$) δ = 7.80 (dd, *J* = 7.9, 1.2 Hz, 1H), 7.57 (dd, *J* = 7.8, 1.7 Hz, 1H), 7.38 (td, *J* = 7.6, 1.3 Hz, 1H) 6.97 (td, *J* = 7.7, 1.8 Hz, 1H), 5.08 (qd, *J* = 6.4, 3.3 Hz, 1H), 1.96 (d, J = 3.3 Hz, 1H), 1.47 (d, *J* = 6.4 Hz, 3H). **^{13}C-NMR (151 MHz, $CDCl_3$)** δ = 147.6, 139.5, 129.3, 128.9, 126.5, 97.4, 73.9, 23.9. **FTIR (ATR, neat)** $\tilde{\nu}$ = 3312, 3057, 2970, 2924, 1584, 1563, 1462, 1435, 1367, 1260, 1199, 1124, 1086, 1067, 1045, 1007, 897, 751, 720, 652. **MS (EI, 70 eV)** *m/z* = 248.0 $[M]^{+\cdot}$, 233.0 $[M\text{-}CH_3]^{+}$. The analytical data is in accordance with literature data.[1]

4 Substrate synthesis

4.1 General Procedures for the One-Pot Reaction Utilizing Primary Benzyl Acetates 3 and Secondary Benzyl Alcohol 4 (OPP1)

4.1.1 Standard conditions employing primary benzyl acetates 3 (OPP1a)

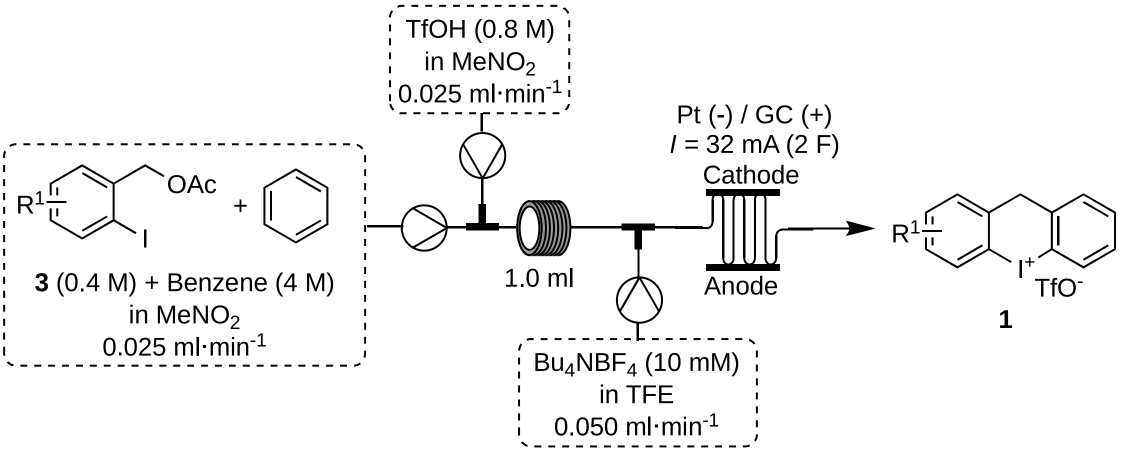

*Scheme S2: General scheme for the synthesis of iodinium salts **1** from primary benzyl acetates **3**.*

The reaction was performed in a continuous flow reactor based on three syringe pumps, one reaction chamber (1.0 ml) at room temperature and an electrochemical Flow Reactor (*Vapourtec - Ion Electrochemical Flow Reactor*, Volume = 0.60 ml, spacer 0.50 mm, 0.10 ml·min^{-1}). All parts were joined with T-Pieces as shown in scheme **S2**. A solution of benzyl acetate **3** (0.4 M, 1.00 equiv) and benzene (4.0 M, 10.0 equiv) in $MeNO_2$ was mixed in flow with a solution of TfOH (0.8 M, 2.00 equiv) in $MeNO_2$ and reacted at rt for 20 min at a combined flowrate of 0.05 ml·min^{-1}. Afterwards a solution of *n*-BuN_4BF_4 (10 mM) in TFE was added to the stream and the flow was subjected to electrolysis by employing a glassy carbon (GC) anode and a platinum cathode. The electrolysis was performed under CCE (j = 2.67 mA·cm^{-2}, 2 F) at a total combined flowrate of 0.10 ml·min^{-1}. The first two reactor volumes were discarded to reach a steady state. After collecting for 3 h 20 min the solution was concentrated by reduced pressure and the product was first precipitated and afterwards washed with Et_2O to obtain the corresponding iodininium salt.

4.1.2 Standard conditions employing secondary benzyl alcohol 4 (OPP1b)

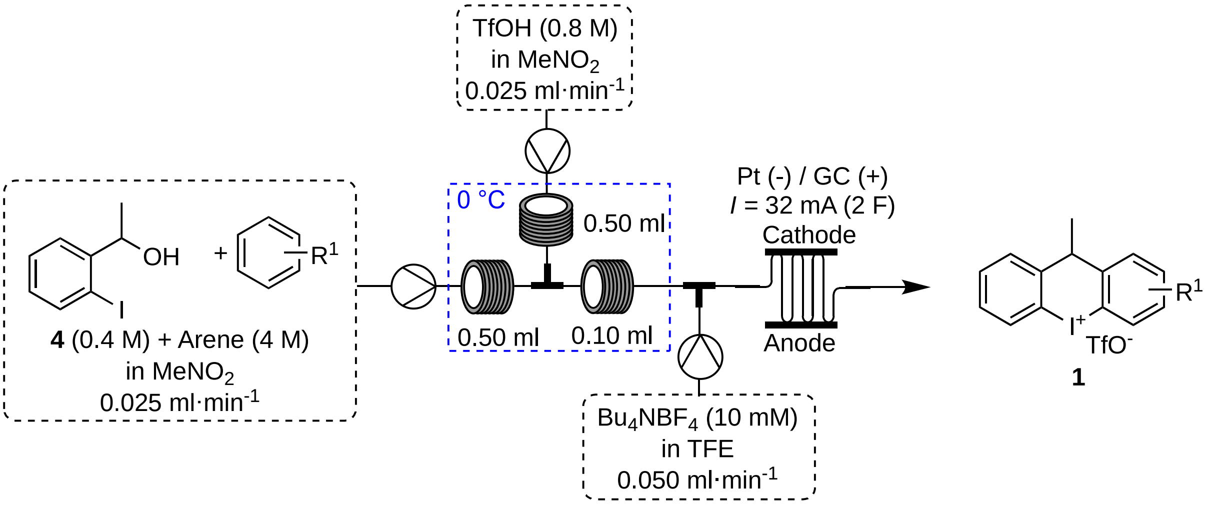

*Scheme S3: General scheme for the synthesis of iodinium salts **1** from secondary benzyl alcohols **4**.*

The reaction was performed in a continuous flow reactor based on three syringe pumps, one reaction chamber (0.10 ml) at room temperature and an electrochemical Flow Reactor (*Vapourtec - Ion Electrochemical Flow Reactor*, Volume = 0.60 ml, spacer 0.50 mm, 0.10 ml·min^{-1}). All parts were joined with T-Pieces as shown in scheme **S3**. An in flow precooled (for 20 min @ 0 °C) solution of benzyl alcohol **4** (0.4 M, 1.00 equiv) and the corresponding arene (4.0 M, 10.0 equiv) in $MeNO_2$ was mixed with an in flow precooled (for 20 min @ 0 °C) solution of TfOH (0.8 M, 2.00 equiv) in $MeNO_2$ and reacted at 0 °C for 2 min at a combined flowrate of 0.05 ml·min^{-1}. Afterwards a solution of *n*-BuN_4BF_4 (10 mM) in TFE was added to the stream and the flow was subjected to electrolysis by employing a glassy carbon (GC) anode and a platinum cathode. The electrolysis was performed under CCE (j = 2.67 mA·cm^{-2}, 2 F) at a total combined flowrate of 0.10 ml·min^{-1}. The first two reactor volumes were discarded to reach a steady state. After collecting for 3 h 20 min the solution was concentrated by reduced pressure and the product was first precipitated and afterwards washed with Et_2O to obtain the corresponding iodininium salt.

4.2 Substrates from Primary Benzyl Acetates and Secondary Benzyl Alcohols

10*H*-Dibenzo[*b,e*]iodinin-5-ium trifluoromethanesulfonate (1a)

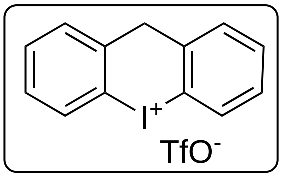

Following **OPP1a**, the reaction of 2-iodobenzyl acetate (**3a**, 552 mg, 2.00 mmol) gave the product **1a** (373 mg, 0.844 mmol, 42%) as a colourless solid.

^{1}H-NMR (601 MHz, DMSO-*d*$_6$) δ = 8.10 (dd, J = 8.1, 1.2 Hz, 2H), 7.81 (dd, J = 7.6, 1.6 Hz, 2H), 7.61 (td, J = 7.5, 1.2 Hz, 2H), 7.43 (td, J = 7.7, 1.6 Hz, 2H), 4.32 (s, 2H). **^{13}C-NMR (151 MHz, DMSO-*d*$_6$)** δ = 138.9, 133.7, 131.8, 130.5, 129.1, 120.7 (q, J = 322.7 Hz), 116.0, 45.7. **^{19}F-NMR (565 MHz, DMSO-*d*$_6$)** δ = -77.7. **FTIR (ATR, neat)** $\tilde{\nu}$ = 3094, 3059, 1457, 1441, 1426, 1237, 1220, 1152, 1023, 751. **MS (ESI$^+$, MeCN/H_2O)** *m/z* = 292.9 [M-OTf]$^+$. **Mp** T = 219 – 221 °C (decomp.). The analytical data is in accordance with literature data.[1]

2-Fluoro-10*H*-dibenzo[*b,e*]iodinin-5-ium trifluoromethanesulfonate (1b)

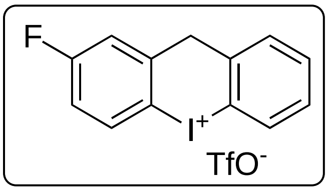

Following **OPP1a**, the reaction of 5-fluoro-2-iodobenzyl acetate (**3b**, 588 mg, 2.00 mmol) gave the product **1b** (135 mg, 0.294 mmol, 15%) as a colourless solid.

^{1}H-NMR (601 MHz, DMSO-*d*$_6$) δ = 8.12 (dd, J = 8.9, 5.3 Hz, 1H), 8.09 (dd, J = 8.1, 1.2 Hz, 1H), 7.77 (dd, J = 7.6, 1.6 Hz, 1H), 7.74 (dd, J = 9.3, 3.0 Hz, 1H), 7.62 (td, J = 7.5, 1.2 Hz, 1H), 7.44 (ddd, J = 8.2, 7.4, 1.6 Hz, 1H), 7.36 (td, J = 8.7, 3.0 Hz, 1H), 4.34 (s, 2H). **^{13}C-NMR (151 MHz, DMSO-*d*$_6$)** δ = 163.8 (d, J = 249.8 Hz), 142.2 (d, J = 8.8 Hz), 138.4, 135.7 (d, J = 9.0 Hz), 133.6, 131.8, 130.6, 129.2, 120.7 (q, J = 322.3 Hz), 117.6 (d, J = 23.6 Hz), 116.3 (d, J = 24.2 Hz), 116.2, 110.1 (d, J = 2.4 Hz), 45.3. **^{19}F-NMR (565 MHz, DMSO-*d*$_6$)** δ = -77.7, -109.3 (td, J = 8.8, 5.3 Hz). **FTIR (ATR, neat)** $\tilde{\nu}$ = 3098, 1573, 1460, 1272, 1220, 1173, 1021, 998, 817, 758. **MS (ESI$^+$, MeCN/H_2O)** *m/z* = 310.9 [M-OTf]$^+$. **Mp** T = 225 – 230 (decomp.). The analytical data is in accordance with literature data.[1]

2-Chloro-10*H*-dibenzo[*b,e*]iodinin-5-ium trifluoromethanesulfonate (1c)

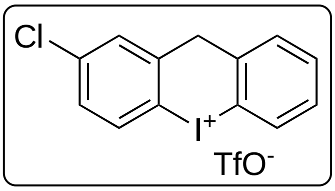

Following **OPP1a**, the reaction of 5-chloro-2-iodobenzyl acetate (**3c**, 621 mg, 2.00 mmol) gave the product **1c** (249 mg, 0.523 mmol, 26%) as a colourless solid.

^{1}H-NMR (601 MHz, DMSO-*d*$_6$) δ = 8.09 (dd, *J* = 8.1, 1.2 Hz, 1H), 8.08 (d, *J* = 8.6 Hz, 1H), 7.95 (d, *J* = 2.5 Hz, 1H), 7.77 (dd, *J* = 7.6, 1.6 Hz, 1H), 7.62 (td, *J* = 7.5, 1.2 Hz, 1H), 7.54 (dd, *J* = 8.6, 2.5 Hz, 1H), 7.44 (td, *J* = 8.1, 1.6 Hz, 1H), 4.33 (s, 2H). **^{13}C-NMR (151 MHz, DMSO-*d*$_6$)** δ = 141.4, 138.4, 136.6, 135.2, 133.6, 131.9, 130.7, 130.1, 129.2, 128.8, 120.7 (q, *J* = 322.2 Hz), 116.2, 114.1, 45.1. **^{19}F-NMR (565 MHz, DMSO-*d*$_6$)** δ = -77.7. **FTIR (ATR, neat)** $\tilde{\nu}$ = 3085, 1556, 1453, 1276, 1220, 1171, 1021, 993, 785, 756. **MS (ESI$^+$, MeCN/H$_2$O)** *m/z* = 344.9 [M+H$_2$O-OTf]$^+$. **Mp** *T* = 216 – 218. The analytical data is in accordance with literature data.[1]

2-Methyl-10*H*-dibenzo[*b,e*]iodinin-5-ium trifluoromethanesulfonate (1d)

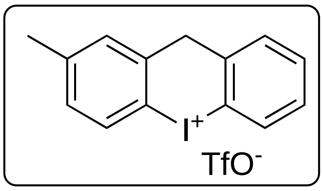

Following **OPP1a**, the reaction of 2-iodo-5-methylbenzyl acetate (**3d**, 580 mg, 2.00 mmol) gave the product **1d** (496 mg, 1.09 mmol, 54%) as a colourless solid.

^{1}H-NMR (600 MHz, DMSO-*d*$_6$) δ = 8.08 (dd, *J* = 8.1, 1.2 Hz, 1H), 7.95 (d, *J* = 8.2 Hz, 1H), 7.79 (dd, *J* = 7.5, 1.6 Hz, 1H), 7.63 (d, *J* = 2.1 Hz, 1H), 7.60 (td, *J* = 7.4, 1.1 Hz, 1H), 7.42 (td, *J* = 7.7, 1.7 Hz, 1H), 7.25 (dd, *J* = 8.3, 2.1 Hz, 1H), 4.26 (s, 2H), 2.37 (s, 3H). **^{13}C-NMR (151 MHz, DMSO-*d*$_6$)** δ = 142.0, 139.0, 138.7, 133.6, 133.3, 131.7, 131.1, 130.5, 129.7, 129.0, 120.7 (q, *J* = 322.2 Hz), 116.0, 112.2, 45.6, 20.6. **^{19}F-NMR (565 MHz, DMSO-*d*$_6$)** δ = -77.7. **FTIR (ATR, neat)** $\tilde{\nu}$ = 3046, 1594, 1460, 1443, 1388, 1277, 1243, 1152, 1025, 746. **HRMS (ESI$^+$, MeOH)** *m/z* = 306.99760 [M-OTf]$^+$. Calculated for [C$_{14}$H$_{12}$I]$^{+\cdot}$: *m/z* = 306.99782. **Mp** *T* = 200 – 205 (decomp.).

3-Bromo-10*H*-dibenzo[*b,e*]iodinin-5-ium trifluoromethanesulfonate (1e)

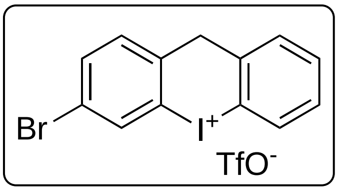

Following **OPP1a**, the reaction of 3-bromo-2-iodobenzyl acetate (**3e**, 710 mg, 2.00 mmol) gave the product **1e** (171 mg, 0.329 mmol, 16%) as a colourless solid.

^{1}H-NMR (600 MHz, DMSO-*d*$_6$) δ = 8.27 (d, *J* = 2.0 Hz, 1H), 8.10 (dd, *J* = 8.1, 1.2 Hz, 1H), 7.83 (dd, *J* = 8.1, 2.0 Hz, 1H), 7.79 (dd, *J* = 7.6, 1.6 Hz, 1H), 7.75 (d, *J* = 8.2 Hz, 1H), 7.62 (td, *J* = 7.5, 1.2 Hz, 1H), 7.44 (td, *J* = 7.7, 1.7 Hz, 1H), 4.30 (s, 2H). **^{13}C-NMR (151 MHz, DMSO-*d*$_6$)** δ = 138.7, 138.5, 135.5, 134.5, 133.7, 131.9, 131.8, 130.6, 129.2, 121.8 (q, *J* = 322 Hz), 120.4, 117.0, 116.2, 44.9. **^{19}F-NMR (565 MHz, DMSO-*d*$_6$)** δ = -77.7. **FTIR (ATR, neat)** $\tilde{\nu}$ = 3097, 1576, 1457, 1379, 1282, 1233, 1159, 1025, 842, 751. **MS (ESI$^+$, MeCN/H$_2$O)** *m/z* = 3790.8 [M-OTf]$^+$. **Mp** *T* = 210 – 215 (decomp.). The analytical data is in accordance with literature data.[1]

4-Methyl-10*H*-dibenzo[*b,e*]iodinin-5-ium trifluoromethanesulfonate (1f)

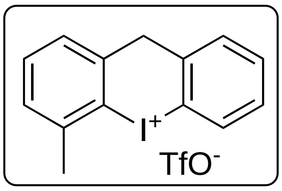

Following **OPP1a**, the reaction of 2-iodo-3-methylbenzyl acetate (**3f**, 580 mg, 2.00 mmol) gave the product **1f** (313 mg, 0.686 mmol, 34%) as a colourless solid.

^{1}H-NMR (600 MHz, DMSO-*d*$_6$) δ 8.10 (dd, *J* = 8.2, 1.1 Hz, 1H), 7.82 (dd, *J* = 7.6, 1.6 Hz, 1H), 7. 64 (d, *J* = 7.4 Hz, 1H), 7.62 (td, *J* = 7.5, 1.2 Hz, 1H), 7.52 (t,

J = 7.4 Hz, 1H), 7.42 (td, *J* = 8.2, 1.6 Hz, 1H), 7.39 (d, *J* = 7.3 Hz, 1H), 4.35 (s, 2H), 2.63 (s, 3H). **^{13}C-NMR (151 MHz, DMSO-*d*$_6$)** δ = 140.6, 139.7, 139.0, 133.7, 131.9, 131.7, 130.3, 129.5, 129.0, 128.0, 120.7 (q, *J* = 322.3 Hz), 120.5, 117.0, 47.2, 24.6 **^{19}F-NMR (565 MHz, DMSO-*d*$_6$)** δ = -77.7. **FTIR (ATR, neat)** $\tilde{\nu}$ = 1460, 1440, 1425, 1275, 1228, 1172, 1021, 994, 810, 745. **HRMS (ESI$^+$, MeOH)** *m/z* = 306.99762 [M-OTf]$^+$. Calculated for $[C_{14}H_{12}I]^{+\cdot}$: *m/z* = 306.99782. **Mp** *T* = 120 – 125 (decomp.).

10-Methyl-10*H*-dibenzo[*b,e*]iodinin-5-ium trifluoromethanesulfonate (1g)

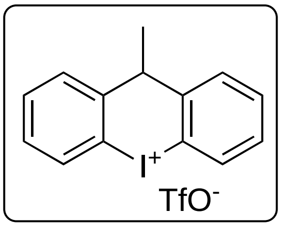

Following **OPP1b**, the reaction of 1-(2-iodophenyl)ethan-1-ol (**4**, 710 mg, 2.00 mmol) with benzene (1.56 g, 1.78 ml, 20.0 mmol) gave the product **1g** (346 mg, 0.759 mmol, 38%) as a colourless solid.

^{1}H-NMR (600 MHz, DMSO-*d*$_6$) δ = 8.13 (dd, *J* = 8.0, 1.2 Hz, 1H), 7.74 (dd, *J* = 7.8, 1.6 Hz, 1H), 7.64 (td, *J* = 7.5, 1.2 Hz, 1H), 7.43 (td, *J* = 7.7, 1.6 Hz, 1H), 4.25 (bs, 1H), 1.75 (bs, 2H). **^{13}C-NMR (151 MHz, DMSO-*d*$_6$)** δ = 141.6, 133.9, 131.8, 129.0, 128.8, 120.6 (q, *J* = 323 Hz), 47.8. Two Signals are missing due to signal broadening. **^{19}F-NMR (565 MHz, DMSO-*d*$_6$)** δ = -77.7. **FTIR (ATR, neat)** $\tilde{\nu}$ = 3257, 3077, 2990, 1589, 1559, 1446, 1362, 1307, 1280, 1260, 1245, 1198, 1178, 1134, 1108, 1081, 1067, 1025, 906, 831, 777, 763, 746. **HRMS (ESI$^+$, MeOH)** *m/z* = 306.99751 [M-OTf]$^+$. Calculated for $[C_{14}H_{12}I]^{+\cdot}$: *m/z* = 306.99782. **Mp** *T* = 255 – 257.

3,10-Dimethyl-10*H*-dibenzo[*b,e*]iodinin-5-ium trifluoromethanesulfonate (1h)

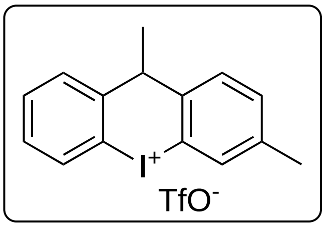

Following **OPP1b**, the reaction of 1-(2-iodophenyl)ethan-1-ol (**4**, 710 mg, 2.00 mmol) with toluene (1.84 g, 2.12 ml, 20.0 mmol) gave the product **1h** (347 mg, 0.738 mmol, 37%) as a colourless solid.

^{1}H-NMR (DMSO-*d*$_6$, 600 MHz) δ = 8.11 (dd, *J* = 8.2, 1.2 Hz, 1H), 7.93 (d, 1H), 7.73 (d, *J* = 6.8 Hz, 1H), 7.63 (td, *J* = 7.5, 1.2 Hz, 1H), 7.62 (d, *J* = 8.0 Hz, 1H), 7.45 (dd, *J* = 7.9, 1.6 Hz, 1H), 7.42 (td, *J* = 7.7, 1.6 Hz, 1H), 4.21 (bs, 1H), 2.36 (s, 3H), 1.72 (bs, 3H). **^{13}C-NMR (DMSO-*d*$_6$, 151 MHz)** δ = 141.8, 138.9, 138.6, 133.9, 133.8, 132.4, 131.8, 129.0, 128.5 (m, 2C), 120.7 (q, *J* = 322.6 Hz), 47.3, 20.2, 16.3. Two Signals are missing due to signal broadening. **^{19}F-NMR (DMSO-*d*$_6$, 565 MHz)** δ = -77.7. **FTIR (ATR, neat)** $\tilde{\nu}$ = 3096, 2976, 2926, 1458, 1438, 1386, 1015, 831, 769, 745. **HRMS (ESI$^+$, MeOH)** *m/z* = 321.01324 [M-TfO]$^+$. Calculated for $C_{15}H_{14}I^{+*}$: *m/z* = 321.01347. **Mp** *T* = 176-178. The analytical data (^{1}H- and ^{13}C-NMR) are in agreement with the by us previously reported corresponding tetrafluoroborate.[1]

4.3 Substrates that could not be successfully synthesized

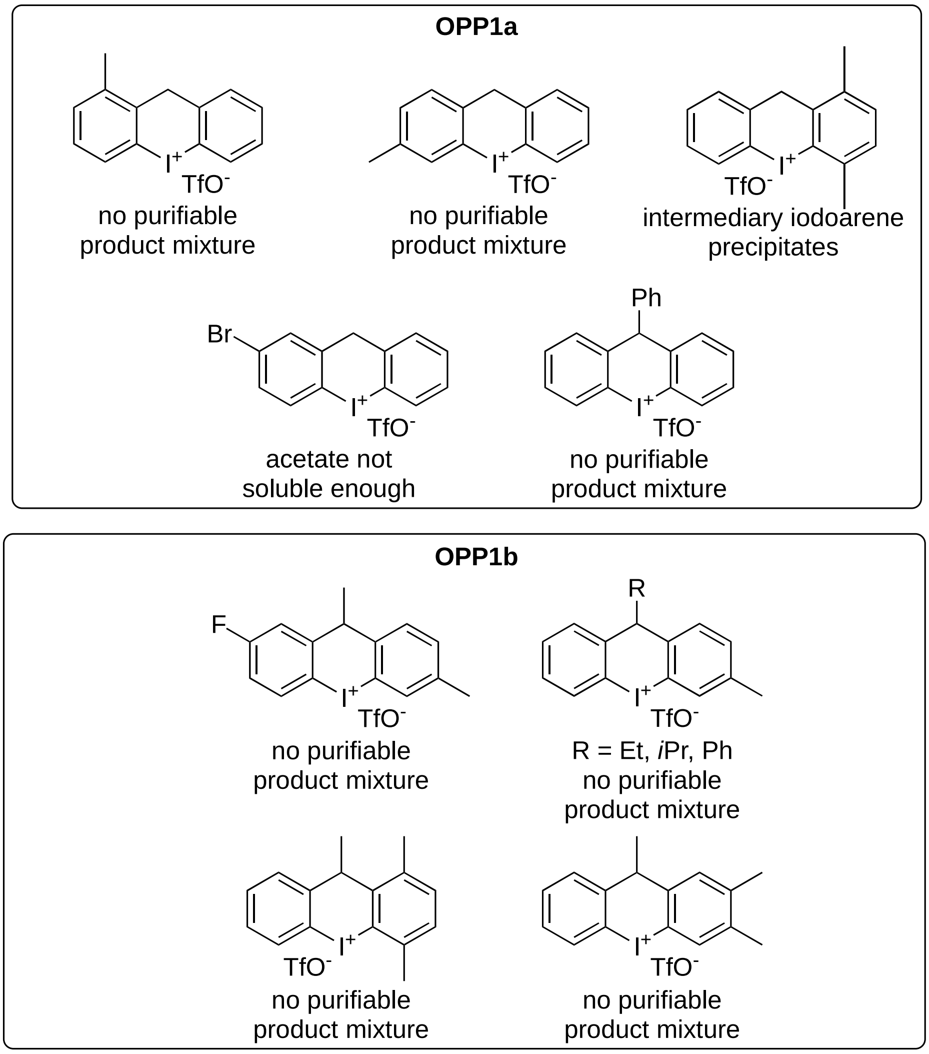

Figure S2: Substrates that could not successfully be synthesized with OPP1a or OPP1b.

5 NMR-Spectra of New Substrates

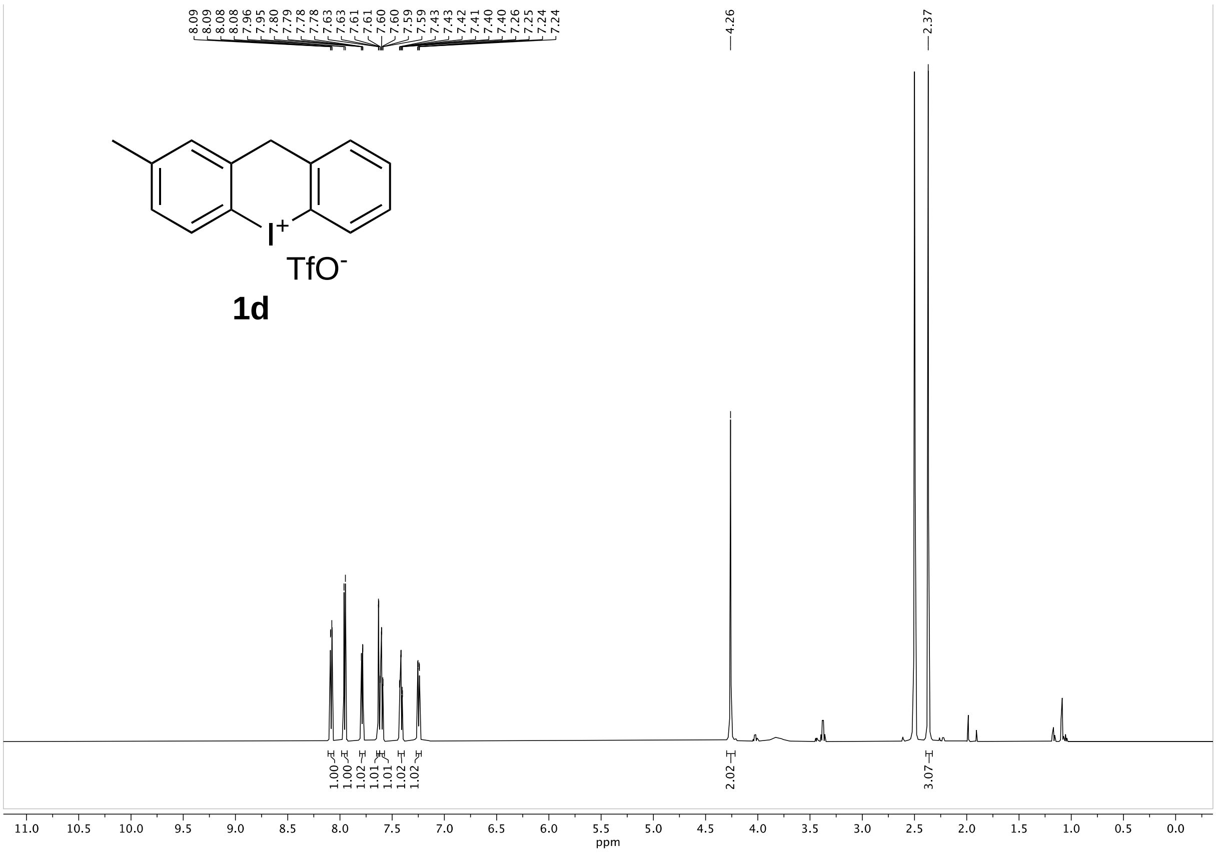

Figure S2: 600 MHz ^{1}H-NMR spectrum of compound **1d**.

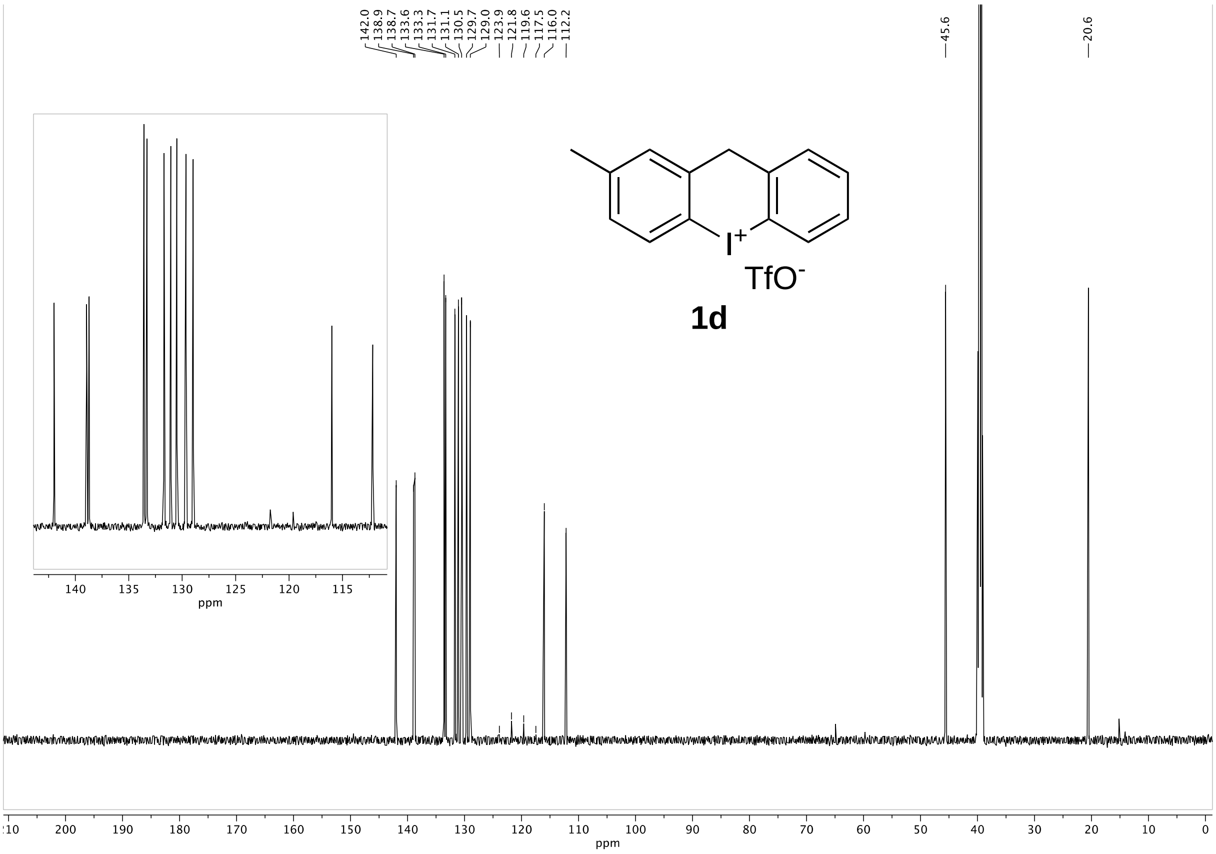

Figure S3: 151 MHz ^{13}C-NMR spectrum of compound **1d**.

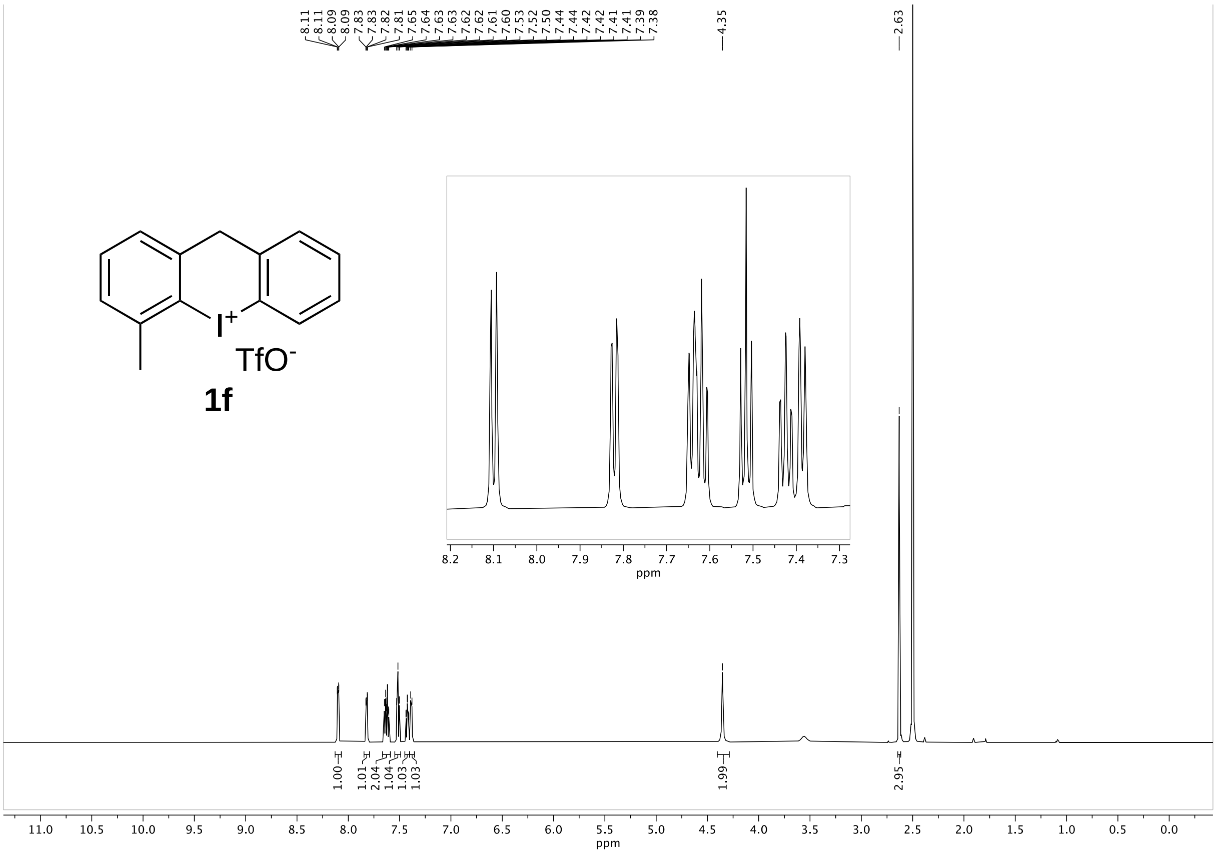

*Figure S4: 600 MHz ^{1}H-NMR spectrum of compound **1f**.*

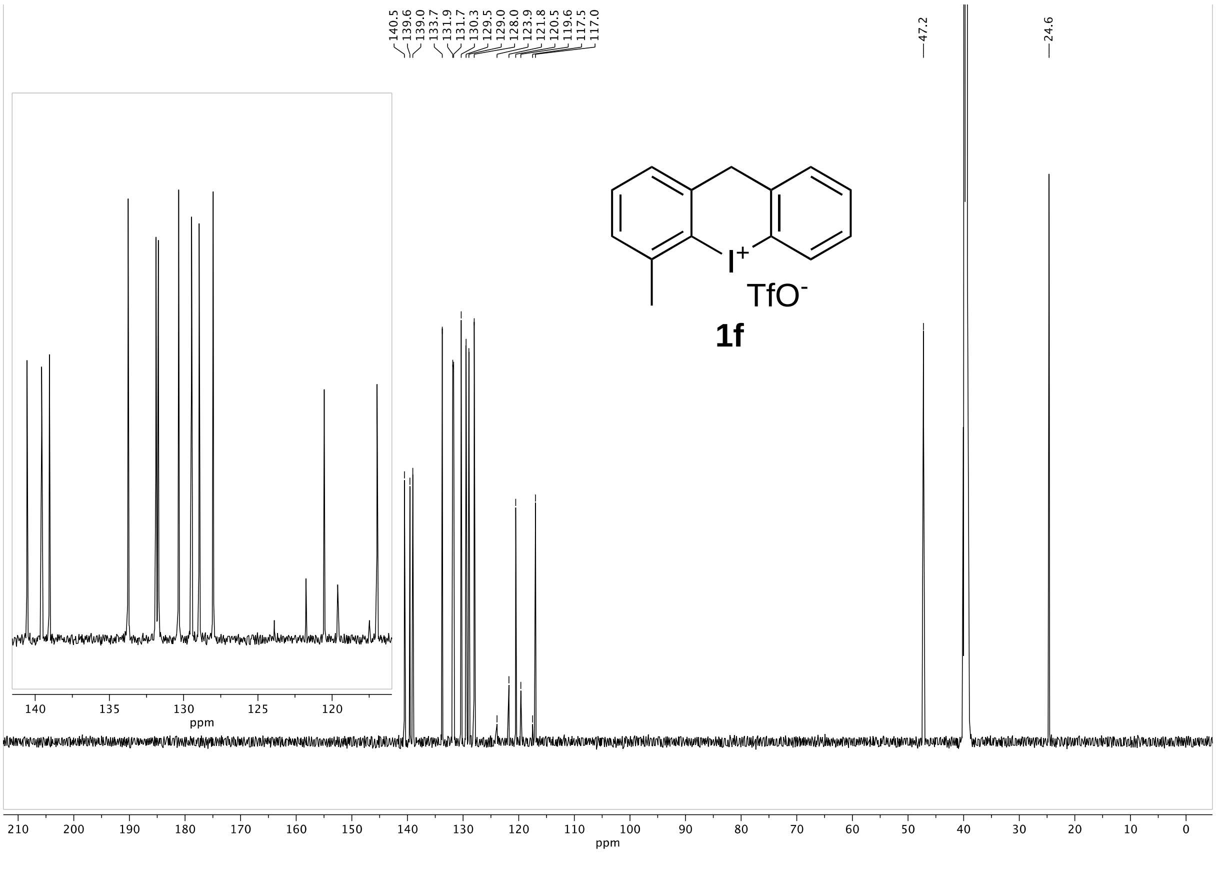

*Figure S5: 151 MHz ^{13}C-NMR spectrum of compound **1f**.*

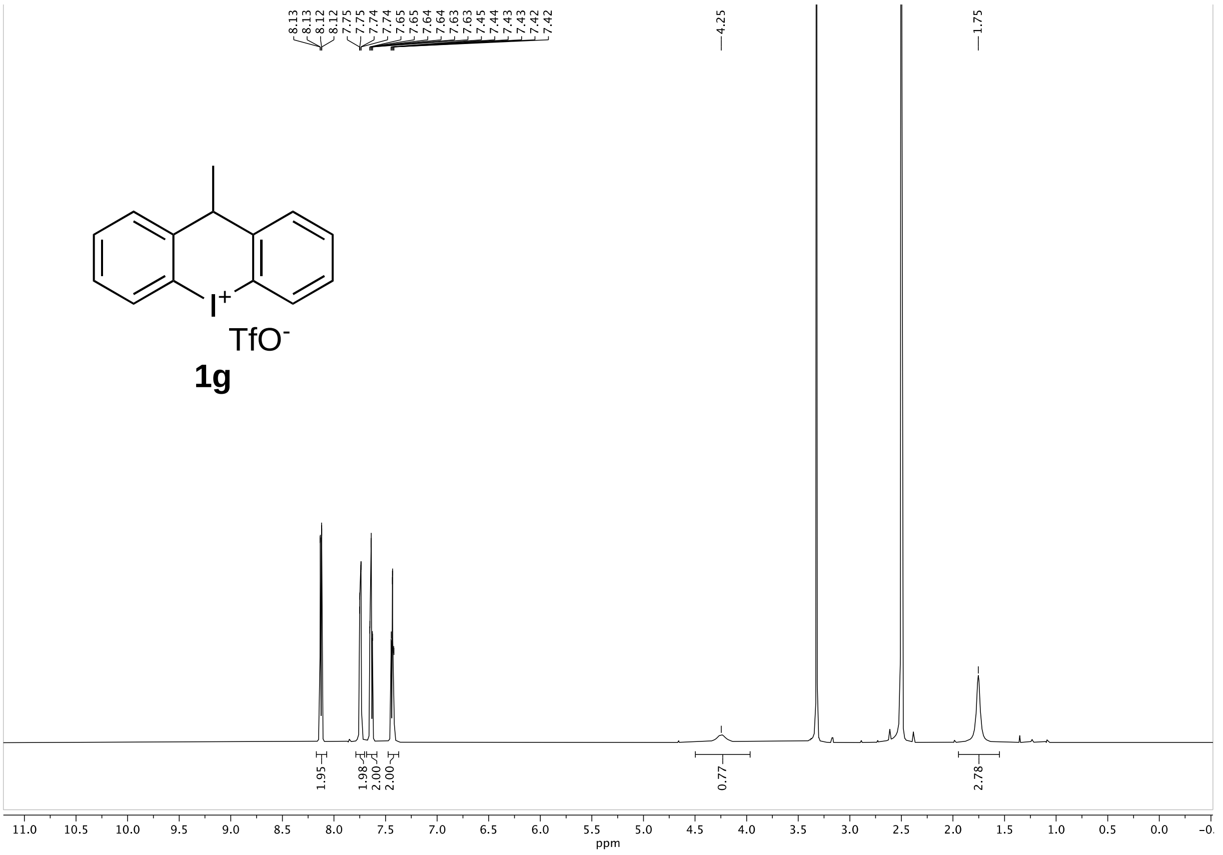

*Figure S6: 600 MHz ^{1}H-NMR spectrum of compound **1g**.*

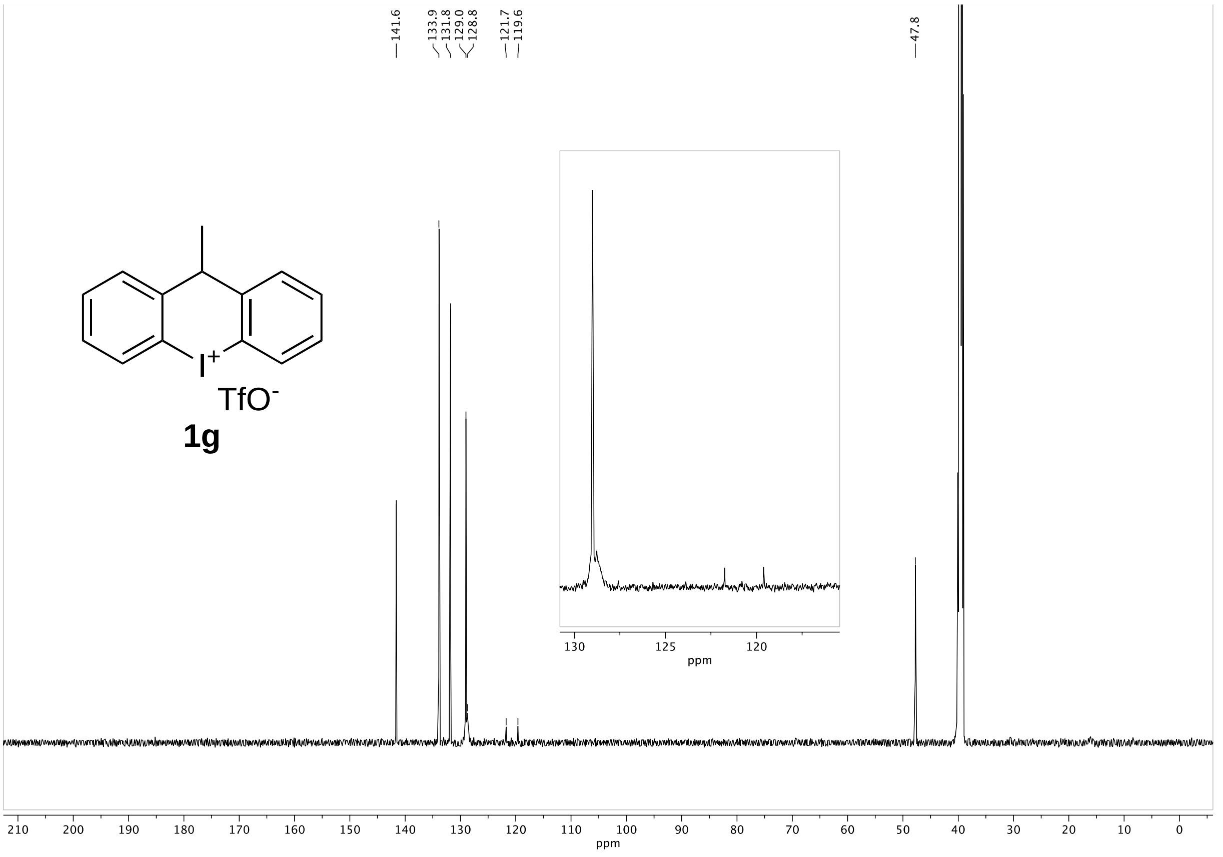

*Figure S7: 151 MHz ^{13}C-NMR spectrum of compound **1g**.*

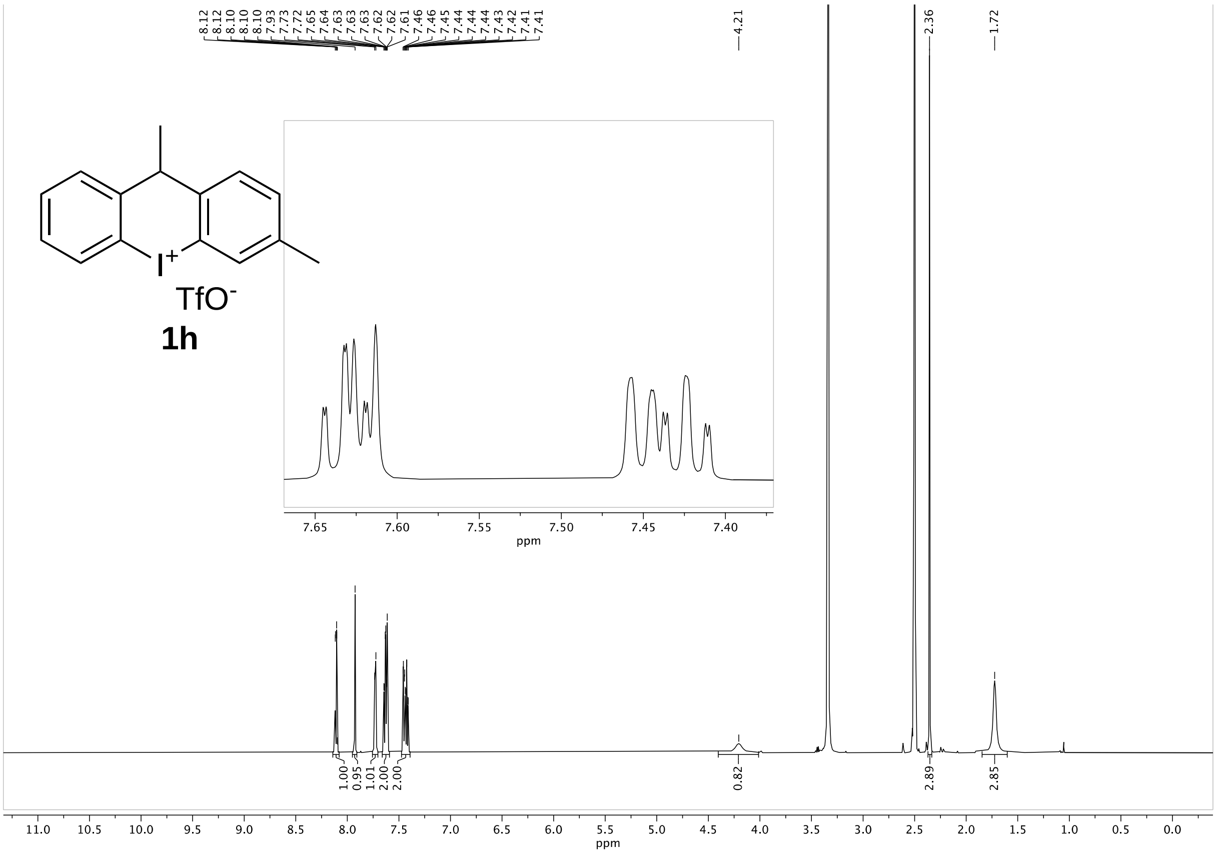

*Figure S8: 600 MHz ^{1}H-NMR spectrum of compound **1h**.*

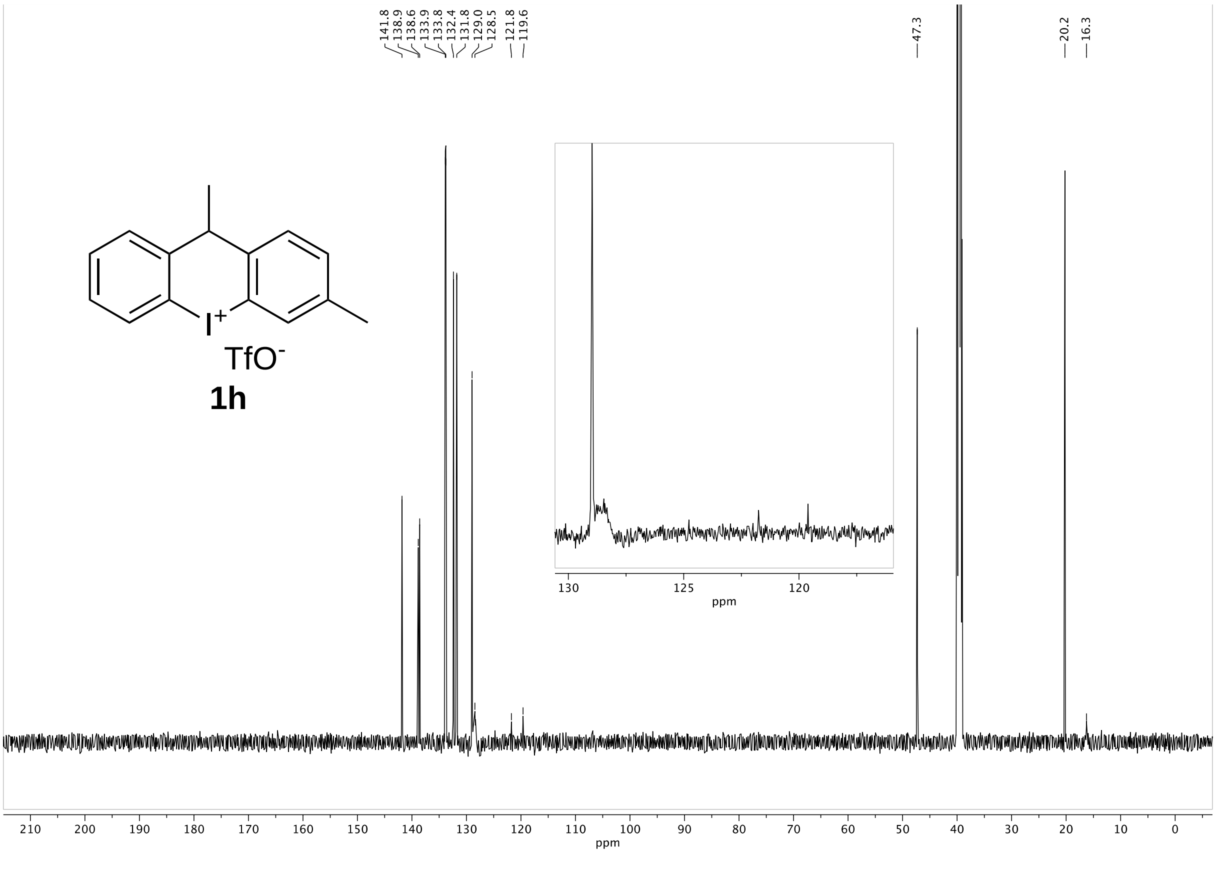

*Figure S9: 151 MHz ^{13}C-NMR spectrum of compound **1h**.*

6 Literature

[1] Caspers, L. D., Spils, J., Damrath, M., Lork, E., Nachtsheim, B. J., *J. Org. Chem.* **2020**, *85*, 9161-9178.

[2] Boelke, A., Nachtsheim, B. J., *Adv. Synth. Catal.* **2019**, *362*, 184-191.

[3] Borah, R., Deka, N., Sarma, J. C., *J. Chem. Res., Synop* . **1997**, 110-111.

[4] Miles, K. C., Le, C. C., Stambuli, J. P., *Chem. - Eur. J.* **2014**, *20*, 11336-11339.

[5] Mei, T. S., Giri, R., Maugel, N., Yu, J. Q., *Angew. Chem. Int. Ed. Engl.* **2008**, *47*, 5215-5219.

[6] Kim, H., Inoue, K., Yoshida, J. I., *Angew. Chem. Int. Ed. Engl.* **2017**, *56*, 7863-7866.

[7] Moorthy, J. N., Senapati, K., Parida, K. N., Jhulki, S., Sooraj, K., Nair, N. N., *J. Org. Chem.* **2011**, *76*, 9593-9601.

[8] Imbos, R., Minnaard, A. J., Feringa, B. L., *J. Am. Chem. Soc.* **2002**, *124*, 184-185.
